# Stable Control of Firing Rate Mean and Variance by Dual Homeostatic Mechanisms

**DOI:** 10.1186/s13408-017-0043-7

**Published:** 2017-01-17

**Authors:** Jonathan Cannon, Paul Miller

**Affiliations:** Brandeis University Department of Biology, Volen National Center for Complex Systems, 415 South St, Waltham, MA 02453 USA

**Keywords:** Homeostasis, Dynamical systems, Stability, Integrator, Synaptic scaling, Averaging, 62P10, 92C20, 37C10, 37C25, 60H10, 34F05, 37E99

## Abstract

Homeostatic processes that provide negative feedback to regulate neuronal firing rates are essential for normal brain function. Indeed, multiple parameters of individual neurons, including the scale of afferent synapse strengths and the densities of specific ion channels, have been observed to change on homeostatic time scales to oppose the effects of chronic changes in synaptic input. This raises the question of whether these processes are controlled by a single slow feedback variable or multiple slow variables. A single homeostatic process providing negative feedback to a neuron’s firing rate naturally maintains a stable homeostatic equilibrium with a characteristic mean firing rate; but the conditions under which multiple slow feedbacks produce a stable homeostatic equilibrium have not yet been explored. Here we study a highly general model of homeostatic firing rate control in which two slow variables provide negative feedback to drive a firing rate toward two different target rates. Using dynamical systems techniques, we show that such a control system can be used to stably maintain a neuron’s characteristic firing rate mean and variance in the face of perturbations, and we derive conditions under which this happens. We also derive expressions that clarify the relationship between the homeostatic firing rate targets and the resulting stable firing rate mean and variance. We provide specific examples of neuronal systems that can be effectively regulated by dual homeostasis. One of these examples is a recurrent excitatory network, which a dual feedback system can robustly tune to serve as an integrator.

## Introduction

Homeostasis, the collection of slow feedback processes by which a living organism counteracts the effects of external perturbations to maintain a viable state, is a topic of great interest to biologists [1, 2]. The brain in particular requires a precise balance of numerous state variables to remain properly operational, so it is no surprise that multiple homeostatic processes have been identified in neural tissues [3]. Some of these processes appear to act at the level of individual neurons to maintain a desirable rate of spiking. When chronic changes in input statistics dramatically lower a neuron’s firing rate, multiple slow changes take place that each act to increase the firing rate again, including the collective scaling of afferent synapses [4, 5] and the adjustment of intrinsic neuronal excitability through adding and removing ion channels [5–7]. These changes suggest the existence of multiple independent slowly-adapting variables that each integrate firing rate over time and provide negative feedback.

Here we undertake an analytical investigation of the dynamics of homeostasis via two independent slow mechanisms (“dual homeostasis”). Our focus on dual homeostasis is partially motivated by the rough breakdown of firing rate homeostatic mechanisms into two categories, synaptic and intrinsic. In our analytical work, we maintain sufficient generality to describe a broad class of firing rate control mechanisms, but we illustrate our results using examples in which homeostasis is governed by one synaptic mechanism acting multiplicatively on neuronal inputs and one intrinsic mechanism acting additively to increase or decrease neuronal excitability. We limit our scope to dual homeostasis to allow us to derive strong analytical results.

It is not immediately clear that dual homeostasis should even be possible. When two variables independently provide negative feedback to drive the same signal toward different targets, one possible outcome is “wind-up” [2], where each variable perpetually ramps up or down in competition with the other to drive the signal toward its own target.

In a recent publication [8], we perform numerical simulations of dual homeostasis (intrinsic and synaptic) in biophysically detailed neurons. We show empirically that this dual homeostasis is stable across a broad swath of parameter space and that it serves to restore not only a characteristic mean firing rate but also a characteristic firing rate variance after perturbations.

Here, we demonstrate analytically that stable homeostasis occurs in a broad family of dual control systems. Further, we find that dual homeostatic control naturally preserves both the mean and the variance of the firing rate, a task impossible for a homeostatic system with a single slow feedback mechanism. We identify broad conditions under which a dually homeostatic neuron possesses a stable homeostatic fixed point, and we derive estimates of the characteristic firing rate mean and variance maintained by homeostasis in terms of homeostatic parameters. We use rate-based neurons and Poisson-spiking neurons for illustration, but our main result is sufficiently general to apply to any model neuron.

One specific application in which such a control system could play an essential role is in tuning a recurrent excitatory network to serve as an integrator. This task is generally considered one that requires biologically implausible precise calibration of multiple parameters [9, 10] and is not well understood (though various solutions to the fine tuning problem have been proposed in [11–14]). In [8], we show empirically that a heterogeneous network of dually homeostatic neurons can tune itself to serve as an integrator. Here, we demonstrate analytically in a simple model of a recurrent excitatory network that integrating behavior can be stabilized by single-cell dual homeostasis and that this stability is robust to the homeostatic parameters of the neurons in the network.

In Sect. 2, we introduce our generalized model of dual homeostasis with the simple but informative example of synaptic/intrinsic firing rate control, and we discuss the reasons that stable homeostatic control is possible for this system. In Sect. 3, we pose a highly general mathematical model of dual homeostatic control. We derive an estimate of the firing rate mean and variance that characterize the fixed points in a given dual homeostatic control system and conditions under which these fixed points are stable. In Sect. 4, we give further specific examples that are encompassed by our general result. In Sect. 5, we use our results to explore dual homeostasis as a strategy for integrator tuning in recurrent excitatory networks. In Sect. 6, we summarize and discuss our results.

## Preliminary Examples

Throughout this manuscript, we consider a homeostatic neuronal firing rate control system with slow homeostatic control variables that serve as parameters for neuronal dynamics. Each of these variables represents a biological mechanism that provides slow negative feedback in response to a more rapidly varying neuronal firing rate *r*. In this section, we focus on an example in which the two control variables are (1) a factor *g* describing the collective scaling of the strengths of afferent synapses and (2) the neuron’s “excitability” *x*, which represents a horizontal shift in the mapping from input current to firing rate. An increase in *x* can be understood as an increase in excitability (or a decrease in firing threshold) and might be implemented in vivo by a change in ion channel density as suggested in [7]. The choice of *x* and *g* as homeostatic control variables is motivated by the observation that synaptic scaling and homeostasis of intrinsic excitability operate concurrently in mammalian cortex [5]. We write this dual control system in the form

1 $$ \begin{aligned} \tau_{{{x}} } \dot{{{x}}} &= f_{{{x}} }(r_{{{x}} }) - f_{{{x}}}(r), \\ \tau_{g } \dot{g} &= g \bigl[f_{g }(r_{g }) - f_{g }(r) \bigr], \end{aligned} $$

where *r* is a neuronal firing rate, $r_{{{x}}}$ and $r_{g }$ are the “target firing rates” of the two homeostatic mechanisms, $f_{{{x}}}$ and $f_{g}$ are increasing functions describing the effect of deviations from the target rates on the two control variables, and $\tau _{{{x}}}$ and $\tau_{g}$ are time constants assumed to be long on the time scale of variation of *r*. An extra factor of *g* multiplies the second ODE because *g* acts as a multiplier and must remain nonnegative. As a result, *g* increases/decreases exponentially (or $\ln(g)$ increases/decreases linearly) if the firing rate *r* is below/above the target rate $r_{g}$. This extra *g* multiplier is not essential for any of the results we derive here.

In general, *r* may represent the firing rate of any type of model neuron, or a correlate of firing rate such as calcium concentration. Likewise, the target rates $r_{{x}}$ and $r_{g}$ may represent firing rates or calcium levels at which the corresponding homeostatic mechanisms equilibrate. We assume that *r* changes quickly relative to $\tau_{{{x}}}$ and $\tau_{g}$ and that *r* is “distribution-ergodic.” This term is defined precisely in the next section; intuitively, it means that over a sufficiently long time, *r* behaves like a series of independent samples from a stationary distribution. This allows us to approximate the right-hand sides in (1) by averages over the distributions of $f_{{x}}(r)$ and $f_{g}(r)$. We will use $\langle\cdot\rangle$ to represent the mean of a stationary distribution. Since the dynamics of the firing rate depends on control variables *x* and *g*, the distributions we consider here also depend on these variables. Averaging (1) over *r*, we can write

2 $$ \begin{aligned} \tau_{{{x}}} \dot{{{x}}} &\approx f_{{{x}}}(r_{{{x}}}) - \bigl\langle f_{{{x}}}(r) \bigr\rangle , \\ \tau_{g} \dot{g} &\approx g \bigl[f_{g }(r_{g }) - \bigl\langle f_{g }(r) \bigr\rangle \bigr]. \end{aligned} $$

In this section, we assume that the neuron is a standard linear firing rate unit with time constant $\tau_{r}$ receiving synaptic input $I{(t)}$. This input is scaled by synaptic strength *g*, and the neuron’s response is additively shifted by the excitability *x*. Thus, the firing rate is described by the equation

3 $$ \tau_{r} \dot{r} = -r + gI{(t)} + {{x}}. $$

In a later section, we will consider a similar system with spiking dynamics.

### Constant Input

**Fig. 1:**
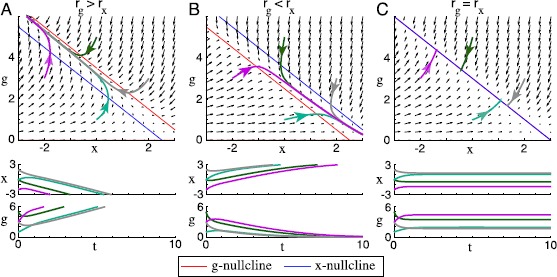
In neurons with constant firing rate, dual homeostasis fails to converge on a set point. A firing rate unit receives constant input $I{(t)} = 1$. It is controlled by the homeostatic *x* (intrinsic homeostasis) and *g* (synaptic homeostasis) as described by (2) with $f_{{x}}(r) = r$ and $f_{g}(r) = r^{2}$. Other parameters are listed in Appendix 2. Vector fields of the control system are illustrated with arrows in the $({{x}}, g)$ phase plane. The *x*- and *g*-nullclines are plotted with sample trajectories in the phase plane (*above*), and these sample trajectories are plotted over time (*below*). (**A**) If the target firing rate $r_{{{x}}}$ of the excitability-modifying homeostatic mechanism is lower than the target firing rate $r_{g }$ of the synaptic scaling mechanism (in this case, $r_{{x}} = 2.5$ and $r_{g} = 3.5$), then *g* increases and *x* decreases without bound. This phenomenon is called “controller wind-up.” (**B**) If $r_{{{x}} } > r_{g }$ (in this case, $r_{{x}} = 2.5$ and $r_{g} = 3.5$), then $g\rightarrow0$, i.e., all afferent synapses are eliminated. (**C**) If $r_{{{x}} } = r_{g }$ (in this case, $r_{{{x}} } = r_{g }=3$), then the nullclines lie on top of each other, creating a one-parameter set of fixed points that collectively attract all control system trajectories

First, let us assume that $I{(t)}$ is equal to a constant *ϕ*. In this case, *r* assumes its asymptotic value $r = g\phi+ {{x}}$ on time scale $\tau_{r}$ and closely tracks this value as *g* and *x* change slowly. Thus, we have $\langle f_{{{x}}}(r)\rangle= f_{{{x}}}(g\phi +{{x}})$ and $\langle f_{g }(r)\rangle= f_{g }(g\phi+{{x}})$. To find the *x*-nullcline (the set of points $({{x}}, g)$ where $\dot {{{x}}} = 0$), we set $\dot{{{x}}} = 0$ in (2). Since $f_{{{x}} }$ is increasing, it is invertible over its range, so we find that the *x*-nullcline in the $({{x}}, g)$ phase plane consists of the set $g\phi+{{x}} = r_{{{x}} }$. Similarly, the *g*-nullcline is the line $g\phi+{{x}} = r_{g }$, plus the set $g = 0$. Fixed points of this ODE are precisely the set of intersections of the nullclines. We are interested primarily in fixed points with $g>0$, so we ignore the set $g = 0$. Representative vector fields, nullclines, and trajectories for (2) with $r = g\phi+ {{x}}$ are illustrated in Fig. 1.

From the nullcline equations, it is clear that if $r_{{{x}} } \neq r_{g }$, there are no fixed points with $g>0$. If $r_{g}>r_{{x}}$, *g* increases and *x* decreases without bound (Fig. 1A); if $r_{g}< r_{{x}}$, then *g* goes to zero (Fig. 1B). Intuitively, this is because the two mechanisms are playing tug-of-war over the firing rate, each ramping up (or down) in a fruitless effort to bring the firing rate to its target. In control theory, this phenomenon is called “wind-up.”

In the (degenerate) case $r_{{{x}} } = r_{g }$, the nullclines overlap perfectly, forming a line of fixed points (Fig. 1C). This situation is undesirable because it leaves an extra degree of freedom: homeostasis has no unique fixed point, so the neuron could reach a set point with any synaptic strength, including arbitrarily strong or weak synapses, depending on initial conditions. Further, this state is destroyed by any perturbation to the target rates, so it could not be easily sustained in a biological system.

These results might lead us to believe that a control system consisting of two homeostatic control mechanisms cannot drive a neuron’s firing rate toward a single stable set-point. However, we shall find that this is only because we have posed the problem in the context of a perfectly constant input $I{(t)}$. When $I{(t)}$ varies, the resulting picture is very different.

### Varying Input

We now consider an input $I{(t)}$ that is not constant at *ϕ*, but instead fluctuates randomly around *ϕ*. One simple example is $I{(t)} = \phi+ \sigma\xi{(t)}$, where $\xi{(t)}$ is white noise with unit variance. In this case, *r* is an Ornstein-Uhlenbeck (OU) process and is described by the stochastic differential equation

4 $$ \tau_{r} \dot{r} = -r +g \bigl(\phi+ \sigma\xi{(t)} \bigr) +{{x}}.$$

An OU process approaches a stationary Gaussian distribution from any initial condition after time $T \gg\tau_{r}$. In this case, this distribution has mean $g\phi+{{x}}$ and variance $\frac{g^{2}\sigma^{2}}{2\tau_{r}}$.

Why does the introduction of variations in $I{(t)}$ change the situation at all? This is closely connected with the basic insight that the mean value of a function *f* over some distribution of arguments *r*, written $\langle f(r)\rangle$, may not be the same as the function *f* applied to the mean of the arguments, written $f(\langle r \rangle )$. The mean value of $f(r)$ is affected by the spread of the distribution of *r* and by the curvature of *f*. Only linear functions *f* have the property that $\langle f(r)\rangle= f(\langle r \rangle)$ for all distributions of *r*.

As a consequence, “satisfying” both homeostatic mechanisms may not require the condition $r_{{{x}} } = \langle r \rangle= r_{g }$. The value of *ẋ* averaged over time may be zero even when the average rate $\langle r\rangle$ is not exactly $r_{{{x}} }$, and the average value of *ġ* may be zero when $\langle r \rangle$ is not exactly $r_{g }$. The conditions required to satisfy each mechanism depend on the entire distribution of *r*, including the mean $\langle r \rangle$ and the variance $\operatorname{var}(r)$. As long as at least one of the homeostatic mechanisms controls $\operatorname{var}(r)$ and $\langle r \rangle$, the system has two degrees of freedom and therefore may satisfy the two fixed-point equations nondegenerately, that is, at a single isolated fixed point.

#### Example 1

Rate model with linear and quadratic feedback

In order to more clearly see the influence of input variation on the control system, we explore the case in which $f_{{{x}} }(r) := r$ and $f_{g }(r) := r^{2}$. Substituting into equation (2) to describe the averaged dynamics of *x* and *g*, we have

$$\begin{aligned} \tau_{{{x}} } \dot{{{x}}} &\approx r_{{{x}} } - \langle r \rangle, \\ \tau_{g } \dot{g} &\approx g \bigl( r_{g }^{2} - \bigl\langle r^{2} \bigr\rangle \bigr). \end{aligned}$$

For the OU process *r*, we have $\langle r \rangle= g\phi+ {{x}}$ and $\langle r^{2} \rangle= \operatorname{var}(r) + \langle r \rangle^{2} = \frac{g^{2}\sigma ^{2}}{2\tau_{r}} + (g\phi+ {{x}})^{2}$, so

5 $$ \begin{aligned} \tau_{{{x}} } \dot{{{x}}} &\approx r_{{{x}} } - (g \phi+ {{x}}), \\ \tau_{g } \dot{g} &\approx g \biggl( r_{g }^{2} - \frac{g^{2}\sigma^{2}}{2\tau _{r}} - (g\phi+ {{x}})^{2} \biggr). \end{aligned} $$

**Fig. 2:**
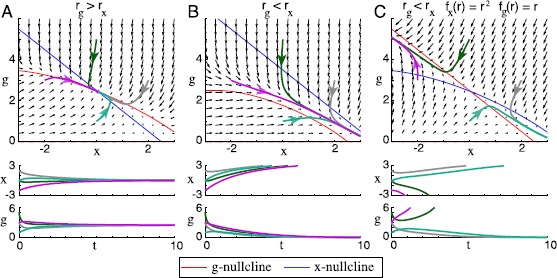
In neurons with fluctuating firing rate, dual homeostasis is effective under certain conditions. A firing rate unit receives a variable synaptic input $I{(t)}$. The parameters are listed in Appendix 2. Vector fields of equation (5) in the $({{x}}, g)$ phase plane are illustrated with arrows. The *x*- and *g*-nullclines are plotted with sample trajectories in the phase plane (*above*), and these sample trajectories are plotted over time (*below*). (**A**) If the target firing rate $r_{{{x}} }$ of the intrinsic homeostatic mechanism is lower than the target firing rate $r_{g }$ of the synaptic scaling mechanism (in this case, $r_{{x}} = 2.5$ and $r_{g} = 3.5$), then the nullclines cross, and all trajectories are attracted to the fixed point at their intersection. (**B**) If $r_{{{x}} } > r_{g }$ (in this case, $r_{{x}} = 3.5$ and $r_{g} = 2.5$), then the nullclines do not cross, and *g* goes to zero. (**C**) If $r_{{{x}} } > r_{g }$ and $f_{{x}}$ is exchanged with $f_{g}$ ($r_{{x}} = 3.5$, $r_{g} = 2.5$, $f_{{x}}(r) = r^{2}$, $f_{g}(r) = r$), then the nullclines do cross, but the resulting fixed point is unstable

A vector field, nullclines, and trajectories for this system are plotted for $\sigma^{2} = 0$ (constant input) in Fig. 1. The same system with $\sigma^{2} = 0.001$ (varying input) is represented in Fig. 2.

The fixed points of this ODE can be studied using basic dynamical systems methods. Setting $\dot{{{x}}} = \dot{g} = 0$, we find that this equation has fixed points at $({{x}}^{*}, g^{*}) = (r_{{{x}} }, 0)$ and $({{x}}^{*}, g^{*}) = (r_{{{x}} } \pm\frac{\phi\sqrt{2\tau_{r}(r_{g }^{2} - r_{{{x}} }^{2})}}{\sigma} , \mp\frac{\sqrt{2\tau_{r} (r_{g }^{2} - r_{{{x}} }^{2})}}{\sigma} )$. We are not interested in the first fixed point because it has $g^{*}=0$. Of the next two fixed points, we are interested only in the one with nonnegative $g^{*}$. This fixed point exists with $g^{*}\neq0$ if and only if the term under the square root is positive, that is, if and only if $r_{g } > r_{{{x}} }$. It is asymptotically stable (i.e., attracts trajectories from all initial conditions in its neighborhood) if the Jacobian of the ODE at the fixed point has two eigenvalues with negative real part. If one or more eigenvalues have positive real part, then it is asymptotically unstable. At this fixed point, the Jacobian is $J =\bigl ( {\scriptsize\begin{matrix}{} -\frac{1}{\tau_{{{x}} }} &-\frac{\phi}{\tau_{{{x}}}} \cr -\frac{2r_{{{x}} } g^{*}}{\tau_{g }} & -\frac{(g^{*}\sigma )^{2}}{\tau_{r}\tau_{g }} - \frac{2r_{{{x}} } \phi g^{*}}{\tau_{g }}\end{matrix}}\bigr ) $, and it is easy to check that both eigenvalues have negative real part. We conclude that this (averaged) system possesses a stable homeostatic set-point *if and only if* $r_{g }>r_{{{x}} }$. At such a fixed point, the firing rate has mean $\langle r \rangle= r_{{{x}} }$ and variance $\langle(r - \langle r \rangle)^{2} \rangle= r_{g }^{2} - r_{{{x}} }^{2}$. Conversely, given any firing rate mean $\mu ^{*}$ and variance $\nu^{*}\ge0$, we can choose targets to stabilize the neuron with this firing rate mean and variance by setting $r_{{{x}} } = \mu^{*}$ and $r_{g } = \sqrt{\nu^{*} + r_{{{x}} }^{2}}$. Note that this equation is *not* dependent on *ϕ* or *σ*, the parameters of the input $I(t)$. Thus, if *ϕ* or *σ* changes, then the homeostatic control system will return the neuron to the same characteristic mean and variance.

In Fig. 2A, $r_{g } > r_{{{x}} }$. In this case, a stable set point exists, and it is evident that it is reached by the following process:

1. If the mean firing rate is below $r_{{{x}} }$, then *g* and *x* increase, both acting to increase the mean firing rate until it is in the neighborhood of $r_{{{x}} }$. If the mean firing rate is above the targets, then *g* and *x* both decrease to lower the mean firing rate to near $r_{{{x}} }$.
2. If *g* is now small, then the firing rate variance $\operatorname {var}(r)$ is small, and the second moment $\langle r^{2} \rangle= \operatorname {var}(r) + \langle r \rangle^{2}$ is close to $r_{{x}}^{2}$. Once $\langle r \rangle$ slightly exceeds $r_{{x}}$, the averaged control system has
  $$\dot{{{x}}} \approx f_{{x}}(r_{{x}}) - \bigl\langle f_{{x}}(r) \bigr\rangle = r_{{x}} - \langle r \rangle< 0, $$
  but $\langle r^{2} \rangle\approx r_{{x}}^{2}$ is still less than $r_{g }^{2}$, so
  $$\dot{g} \approx f_{g}(r_{g}) - \bigl\langle f_{g}(r) \bigr\rangle = r_{g }^{2} - \bigl\langle r^{2} \bigr\rangle > 0. $$
  Alternatively, if *g* is large, then $\operatorname{var}(r)$ is large, so the second moment $\langle r^{2} \rangle$ exceeds $r_{g }^{2}$, whereas $\langle r \rangle$ is still below $r_{{x}}$, *ġ* is negative, and *ẋ* is positive.
3. *g* slowly seeks out the intermediate point between these extremes, where the variance of *r* makes up the difference between $r_{{{x}} }$ and $r_{g }$. As it does so, *x* changes in the opposite direction to keep the mean firing rate near $r_{{{x}} }$.

In Fig. 2B, it is evident that when $r_{g }< r_{{{x}} }$, no such equilibrium exists. In Fig. 2C, we show that if we exchange $f_{{x}}$ with $f_{g}$ and $\tau_{{x}}$ with $\tau_{g}$ such that *g* is linearly controlled and *x* is quadratically controlled, then an equilibrium exists for $r_{g }< r_{{{x}} }$, but it is not stable. These observations suggest that certain general conditions must be met for there to exist a stable equilibrium in a dually homeostatic system. We explore these conditions in the next section.

Note that if only *x* were dynamic but not *g*, the firing rate variance $\operatorname{var}(r)$ would be fixed at $\frac{g^{2}\sigma ^{2}}{2\tau_{r}}$, and therefore the variance at equilibrium would be sensitive to changes in *σ*, the variance of the input current. If only *g* were dynamic but not *x*, the firing rate mean and variance could both change over the course of *g*-homeostasis, but the two would be inseparably linked: using the expressions above for firing rate mean and variance, we can see that no matter how *g* changed we would always have $\langle r \rangle= \frac{\phi\sqrt{2\tau_{r} \operatorname{var}(r)}}{\sigma } + {{x}}$. Thus, the neuron could only maintain a characteristic firing rate mean and variance if they satisfied this constraint.

## Analysis

Now we shall consider the general case in which two control variables *a* and *b* evolve according to arbitrary control functions $f_{a}$ and $f_{b}$ and control the distribution of a neuron’s firing rate *r*. We make the dependence of this distribution on *a* and *b* explicit by writing the distribution of *r* as $P(r ; a,b)$. We address several questions to this model. First, what fixed points exist for a given control system, and what characterizes these fixed points? Second, under what circumstances are these fixed points stable?

In this section, we answer these questions under the simplifying assumption that $f_{a}''(r)$ and $f_{b}''(r)$ are constant on any domain where $P(r ; a,b) > 0$. In Appendices 1 and 2, we show that our results persist qualitatively for nonconstant $f_{a}''$ and $f_{b}''$.

In Theorem 1, we write expressions for the firing rate mean $\mu^{*}$ and variance $\nu^{*}$ that characterize any fixed point $(a^{*}, b^{*})$. From this result we find that the difference between the two target firing rates plays a key role in establishing the characteristic variance at a control system fixed point.

In Theorem 2, we present a general condition that ensures that a fixed point $(a^{*}, b^{*})$ of the averaged control system is stable. This condition takes the form of a relationship between convexity of the control functions and the derivatives of the first and second moments of $P(r;a,b)$ with respect to *a* and *b*.

### Definitions

Consider a pair of homeostatic variables *a* and *b* whose instantaneous rates of change are functions of a firing rate variable *r*:

6 $$ \begin{aligned} \frac{\tau_{a}}{\epsilon} \dot{a} &=f_{a}(r_{a}) - f_{a}(r), \\ \frac{\tau_{b}}{\epsilon} \dot{b} &=f_{b}(r_{b}) - f_{b}(r), \end{aligned} $$

where $0<\epsilon\ll1$ is a multiplier separating the fast time scale of firing rate variation and the slow time scale of homeostasis, $\tau _{a}$ and $\tau_{b}$ are homeostatic time constants (in units of slow time), $r_{a}$ and $r_{b}$ are the target firing rates of the two homeostatic mechanisms, and $f_{a}$ and $f_{b}$ are smooth increasing bounded functions with bounded derivatives. Note that we have introduced the small parameter *ϵ* representing the separation of the time scales of homeostasis and firing rate dynamics rather than assuming that $\tau_{a}$ and $\tau_{b}$ are large. This form is sufficiently general to encompass a wide range of different feedback strategies.

#### Remark 1

In order to describe the evolution of a homeostatic variable *a* that acts multiplicatively and must remain positive (e.g., the synaptic scaling multiplier *g* used in many of our examples), we can instead set $\frac{\tau_{a}}{\epsilon} \dot{a} = a (f_{a}(r_{a}) - f_{a}(r) )$. We can then put this system into the general form above by replacing *a* with $\tilde{a}:=\log(a)$, whose evolution is described by the ODE $\frac{\tau_{a}}{\epsilon}\dot{\tilde{a}} = f_{a}(r_{a}) - f_{a}(r)$.

We assume that, for fixed *a* and *b*, the firing rate $r(t;a,b)$ (written as a function of time and control variables) is distribution-ergodic with stationary firing rate distribution $P(r;a,b)$, that is, $\lim_{T\rightarrow\infty} \frac{1}{T}\int_{0}^{T} f(r(t;a,b)) \, dt = \int_{\mathbb{R}} f(r) P(r;a,b) \,dr$ with probability 1 for all integrable functions *f*. For brevity of notation, we let $\langle\cdot\rangle_{(a,b)} := \mathbb{E}(\cdot| a,b)$ denote the expected value of a function of *r* over the stationary distribution $P(r;a,b)$ (or, equivalently, the time average of this function over time $T\rightarrow\infty$), given a control system state $(a,b)$. Let $\mu{(a,b)}$ and $\nu{(a,b)}$ denote the mean and variance of $P(r;a,b)$, respectively.

Averaging (6) over the invariant distribution, we arrive at the “averaged equations”:

7 $$ \begin{aligned} \frac{\tau_{a}}{\epsilon} \dot{a} &\approx F_{a}(a,b) := f_{a}(r_{a}) - \bigl\langle f_{a}(r) \bigr\rangle _{(a,b)}, \\ \frac{\tau_{b}}{\epsilon} \dot{b} &\approx F_{b}(a,b) := f_{b}(r_{b}) - \bigl\langle f_{b}(r) \bigr\rangle _{(a,b)}. \end{aligned} $$

We use the averaged equations to study the behavior of the unaveraged system (6). Since *r* may be constantly fluctuating, *a* and *b* may continue to fluctuate even once a fixed point of the averaged system has been reached, so we cannot expect stable fixed points in the classical sense. Instead, we define a weaker form of stability.

We shall call a point $(a^{*}, b^{*})$ “stable in the small-*ϵ* limit” if there exists a continuous increasing function *α* with $\alpha(0) = 0$ and an $\epsilon^{*}>0$ such that for all $0<\epsilon <\epsilon^{*}$, the $(a,b)$ trajectory initialized at $(a^{*}, b^{*})$ remains within a radius-$\alpha(\epsilon)$ ball centered at $(a^{*}, b^{*})$ for all time with probability 1. Intuitively, a point is stable in the small-*ϵ* limit if trajectories become trapped in a ball around that point, and the ball is smaller when homeostasis is slower.

#### Lemma 1

*Any exponentially stable fixed point of the averaged system* (7) *is a stable fixed point of the original system* (6) *in the small*-*ϵ* *limit*.

#### Proof

This follows from Theorem 10.5 in [15]. □

### Main Results

#### Fixed Points

Given a homeostatic control state $(a^{*}, b^{*})$, it is straightforward to find the target firing rates that make that state a fixed point in terms of the average values of $f_{a}$ and $f_{b}$. By setting $\dot{a} = \dot{b} = 0$ in (7) we find that $r_{a} = f_{a}^{-1} (\langle f_{a}(r)\rangle_{(a^{*},b^{*})} )$ and $r_{b} = f_{b}^{-1} (\langle f_{b}(r)\rangle_{(a^{*},b^{*})} )$. (These expressions are well defined because $f_{a}$ is increasing and hence invertible, and $\langle f_{a}(r)\rangle_{(a^{*},b^{*})}$ must fall within the range of $f_{a}$; likewise for *b*.)

Given a pair of target firing rates $r_{a}$ and $r_{b}$ and functions $f_{a}$ and $f_{b}$, we can ask what states $(a^{*}, b^{*})$ become fixed points of the averaged system. We shall answer this question in order to show that (1) when $f_{a}''$ and $f_{b}''$ are constant, the fixed points are exactly the points at which $P(r;a,b)$ attains a certain characteristic mean and variance, (2) the relative convexities of the control functions determine whether $r_{a}$ or $r_{b}$ must be larger for fixed points to exist, and (3) fixed points with high firing rate variance are achieved by setting $r_{b}$ far from $r_{a}$.

##### Theorem 1

*Consider a dual control system as described in Sect*. 3.1 *with target firing rates* $r_{a}$ *and* $r_{b}$ *and control functions* $f_{a}$ *and* $f_{b}$. *Let* $K_{a} := \frac{f_{a}''(r_{a})}{f_{a}'(r_{a})}$, $K_{b} := \frac {f_{b}''(r_{b})}{f_{b}'(r_{b})}$, *and* $k := \frac {K_{a}+K_{b}}{K_{a}-K_{b}-K_{a}K_{b}(r_{b}-r_{a})}$. *We consider a domain of control system states* $(a,b)$ *on which each distribution* $P(r; a, b)$ *has constant* $f_{a}''(r)$ *and* $f_{b}''(r)$ *on its support*. *The fixed points of the averaged control system in this domain are exactly the points* $(a^{*}, b^{*})$ *at which the mean* $\mu(a^{*},b^{*})$ *is* $\mu^{*}$ *and the variance* $\nu(a^{*}, b^{*})$ *is* $\nu^{*}$, *where we define*

$$\begin{aligned} \mu^{*} &:= \frac{r_{a}+r_{b}}{2} + \frac{r_{b}-r_{a}}{2}k, \\ \nu^{*} &:= \frac{r_{b} - r_{a}}{K_{b}-K_{a}} \biggl(2 - \frac{r_{b}-r_{a}}{4} \bigl((K_{b} - K_{a}) \bigl(1+k^{2} \bigr) - 2(K_{a}+K_{b})k \bigr) \biggr). \end{aligned}$$

We will henceforth call $\mu^{*}$ and $\nu^{*}$ the “characteristic” mean and variance of any neuron regulated by this control system.

##### Remark 2

Note that this result is *a*/*b* symmetric. If *a* and *b* are swapped, then the signs of $r_{b} - r_{a}$, $K_{b} - K_{a}$, and *k* reverse; however, since these terms all occur in pairs, this reversal leaves the expressions for $\mu^{*}$ and $\nu^{*}$ unchanged.

##### Remark 3

In Appendix 1, we show that this result persists in some sense for nonconstant $f_{a}''$ and $f_{b}''$. Specifically, if variation in $f_{a}''$ and $f_{b}''$ over the appropriate domain is small, the mean and variance at any fixed point are close to $\mu^{*}$ and $\nu^{*}$, and every point at which the mean is $\mu^{*}$ and the variance is $\nu^{*}$ is close to a fixed point.

##### Proof

We abbreviate $\mu{(a,b)}$ as *μ* and $\nu{(a,b)}$ as *ν*. Since $f_{a}$ and $f_{b}$ have constant second derivatives on the domain of interest, we can write

$$ f_{a}(r) = f_{a}(r_{a}) + f'_{a}(r_{a}) (r - r_{a}) + \frac {1}{2}f''_{a}(r_{a}) (r - r_{a})^{2}. $$

Taking the expected values of both sides, we have

$$\bigl\langle f_{a}(r) \bigr\rangle _{(a,b)} = f_{a}(r_{a}) + f'_{a}(r_{a}) (\mu- r_{a}) + \frac{1}{2}f''_{a}(r_{a}) \bigl\langle (r - r_{a})^{2} \bigr\rangle _{(a,b)}. $$

A simple calculation gives us $\langle(r - r_{a})^{2}\rangle _{(a,b)} = \nu+ (\mu-r_{a})^{2}$, so we can write

$$\bigl\langle f_{a}(r) \bigr\rangle _{(a,b)} = f_{a}(r_{a}) + f'_{a}(r_{a}) (\mu- r_{a}) + \frac{1}{2}f''_{a}(r_{a}) \nu+ \frac{1}{2}f''_{a}(r_{a}) (\mu-r_{a})^{2}. $$

At a fixed point $(a^{*}, b^{*})$ of the averaged control system with firing rate mean $\mu^{*}$ and variance $\nu^{*}$, we have $0 = \dot {a} = f_{a}(r_{a}) - \langle f_{a}(r) \rangle_{(a^{*},b^{*})}$, or

8 $$ \begin{aligned} 0 &= f'_{a}(r_{a}) \bigl(\mu^{*} - r_{a} \bigr) + \frac{1}{2}f''_{a}(r_{a}) \nu^{*} + \frac {1}{2}f''_{a}(r_{a}) \bigl(\mu^{*}-r_{a} \bigr)^{2}, \\ 0&=K_{a}\nu^{*} +2 \bigl(\mu^{*} - r_{a} \bigr) + K_{a} \bigl(\mu^{*}-r_{a} \bigr)^{2}. \end{aligned} $$

Deriving a similar expression by expanding $f_{b}(r)$ around $r_{b}$, we have

9 $$ 0=K_{b}\nu^{*} +2 \bigl(\mu^{*} - r_{b} \bigr) + K_{b} \bigl(\mu^{*}-r_{b} \bigr)^{2}. $$

Multiplying (8) by $K_{b}$ and (9) by $K_{a}$ and taking the difference, the $\nu^{*}$ terms cancel, leaving

$$0 = 2K_{b} \bigl(\mu^{*} - r_{a} \bigr)- 2K_{a} \bigl(\mu^{*} - r_{b} \bigr) + K_{a}K_{b} \bigl( \mu^{*}-r_{a} \bigr)^{2} - K_{a}K_{b} \bigl(\mu^{*}-r_{b} \bigr)^{2}. $$

Solving for $\mu^{*} $, we have

$$\mu^{*} = \frac{2K_{a}r_{b} - 2K_{b}r_{a} + K_{a}K_{b}(r_{a}-r_{b})(r_{a}+r_{b})}{2(K_{a}-K_{b}) + 2K_{a}K_{b}(r_{a}-r_{b})} $$

or

$$\begin{aligned} \mu^{*} &= \frac{r_{a}+r_{b}}{2} + \frac{r_{b}-r_{a}}{2}\frac {K_{a}+K_{b}}{K_{a}-K_{b}-K_{a}K_{b}(r_{b}-r_{a})} \\ &= \frac{r_{a}+r_{b}}{2} + \frac{r_{b}-r_{a}}{2}k, \end{aligned}$$

where $k = \frac{K_{a}+K_{b}}{K_{a}-K_{b}-K_{a}K_{b}(r_{b}-r_{a})}$. Taking the difference of (8) and (9), we get

$$0=(K_{a}- K_{b})\nu^{*} +2(r_{b} - r_{a}) + K_{a} \bigl(\mu^{*}-r_{a} \bigr)^{2} - K_{b} \bigl(\mu^{*}-r_{b} \bigr)^{2} $$

or, substituting for $\mu^{*}$ and solving for $\nu^{*}$,

$$\nu^{*} = \frac{(r_{b} - r_{a})}{K_{b}-K_{a}} \biggl(2 - \frac{r_{b}-r_{a}}{4} \bigl((K_{b} - K_{a}) \bigl(1+k^{2} \bigr) - 2(K_{a}+K_{b})k \bigr) \biggr). $$

 □

Given the parameters of the control system (including a pair of target firing rates), this theorem shows that achieving a specific firing rate mean and variance is necessary and sufficient for the time-averaged control system to reach a fixed point. If $P(r;a,b)$ (the distribution of the firing rate as a function of *a* and *b*) changes, as it might as a result of changes in the statistics of neuronal input, then the new fixed points will be the new points at which this firing rate mean and variance are achieved. Conversely, given a desirable firing rate mean and variance, we could tune the parameters of the control system to make these the characteristic mean and variance of the neuron at control system fixed points.

Whether any fixed point $(a^{*}, b^{*})$ actually exists depends on whether the characteristic firing rate mean and variance demanded by Theorem 1 can be achieved by the neuron, that is, fall within the range of $\mu (a^{*},b^{*})$ and $\nu(a^{*}, b^{*})$. If the mapping from $(a, b)$ to $(\mu, \nu)$ is not degenerate, then there exists a nondegenerate (two-parameter) set of reachable values of *μ* and *ν* for which control system fixed points exist. In the degenerate case that neither *μ* nor *ν* depend on *b*, the set of reachable values of *μ* and *ν* are a degenerate one-parameter family in a two-dimensional space. This corresponds to the case of a single-mechanism control system. In this case, a control system possesses a fixed point with a given firing rate mean and variance only if they are chosen in a particular relationship to each other. A perturbation to neuronal parameters would displace this one-parameter family in the $(\mu, \nu)$-space, likely making the preperturbation firing rate mean and variance unrecoverable.

We now prove a corollary giving a simpler form of Theorem 1, which holds if $r_{b} - r_{a}$ is sufficiently small.

##### Corollary 1

*Given* $K_{a}$ *and* $K_{b}$, *let* $K = \max( \lvert K_{a} \rvert, \lvert K_{b} \rvert )$. *If* $r_{a}$ *and* $r_{b}$ *are chosen such that* $K \lvert r_{b}-r_{a} \rvert$ *and* $\frac{K^{2}}{ \lvert K_{b} - K_{a} \rvert} \lvert r_{b} - r_{a} \rvert$ *are sufficiently small*, *then the characteristic mean and variance given in Theorem* 1 *are arbitrarily well approximated by*

$$\begin{aligned} \mu^{*} &\approx\frac{r_{a}+r_{b}}{2} - \frac{r_{b}-r_{a}}{2}\frac {K_{a}+K_{b}}{K_{b}-K_{a}}, \\ \nu^{*} &\approx2\frac{r_{b} - r_{a}}{K_{b}-K_{a}}. \end{aligned}$$

##### Proof

For *k* defined in Theorem 1, we can write

$$k = \frac{K_{a}+K_{b}}{(K_{a}-K_{b}) (1-\frac {K_{a}K_{b}}{K_{a}-K_{b}}(r_{b}-r_{a}) )}, $$

so that if $\frac{K^{2}}{ \lvert K_{b} - K_{a} \rvert} \lvert r_{b} - r_{a} \rvert$ is sufficiently small, then *k* is arbitrarily close to $\frac {K_{a}+K_{b}}{K_{a}-K_{b}}$. This gives us an approximation of $\mu^{*}$. We can also use it to write

$$\begin{aligned} & \biggl\lvert \frac{r_{b}-r_{a}}{4} \bigl((K_{b} - K_{a}) \bigl(1+k^{2} \bigr) - 2(K_{a}+K_{b})k \bigr) \biggr\rvert \\ &\quad\approx \biggl\lvert \frac{1}{4} \biggl((K_{b} - K_{a}) (r_{b}-r_{a})+ \frac{K_{a}^{2} + 2K_{a}K_{b} +K_{b}^{2}}{K_{a}-K_{b}}(r_{b} - r_{a}) \\ &\qquad {}- 2\frac{K_{a}^{2} + 2K_{a}K_{b} +K_{b}^{2}}{K_{a}-K_{b}}(r_{b} - r_{a}) \biggr) \biggr\rvert . \end{aligned}$$

All of these terms are bounded in norm by multiples of either $K \lvert r_{b}-r_{a} \rvert$ or $\frac{K^{2}}{ \lvert K_{b} - K_{a} \rvert} \lvert r_{b} - r_{a} \rvert$, so this expression is arbitrarily small. This gives us an approximation for $\nu^{*}$. □

The range of $r_{b} - r_{a}$ for which this result holds is determined by $K_{a}$ and $K_{b}$, measures of the convexities of the control functions. Informally, we say that this corollary holds if $r_{b} - r_{a}$ is “small on the scale of the convexity of the control functions.”

From the corollary we draw two important conclusions that hold while $r_{b} - r_{a}$ remains small on the scale of the convexity of the control functions:

1. Since a negative firing rate variance can never be achieved by the control system, there can only be a fixed point if $r_{b} - r_{a}$ and $K_{b} - K_{a}$ take the same sign.
2. Increasing $\lvert r_{b} - r_{a} \rvert$ causes a proportionate increase in control system’s characteristic firing rate variance.

#### Fixed Point Stability

Next, we address the question of whether a fixed point of the averaged control system is stable. We again make the simplifying assumption that $f''_{a}$ and $f''_{b}$ are constant and then drop this assumption in Appendix 2.

##### Theorem 2

*Let* $(a^{*}, b^{*})$ *denote a fixed point of the averaged control system described above*. *We assume the following*:

1. *The functions* *μ* *and* *ν* *are differentiable at* $(a^{*}, b^{*})$.
2. $\frac{\partial F_{a}}{\partial a}$ *and* $\frac{\partial F_{b}}{\partial b} $ *are negative at* $(a^{*}, b^{*})$, *that is*, *on average*, *a* *and* *b* *provide negative feedback to* *r* *near* $(a^{*}, b^{*})$.
3. *For* $(a,b)$ *in a neighborhood of* $(a^{*}, b^{*})$, $f''_{a}$ *and* $f''_{b}$ *are constant on any domain of* *r* *where* $P(r; a, b)>0$.

*Let* $\mu^{*} = \mu(a^{*}, b^{*})$ *and* $\nu^{*} = \nu(a^{*}, b^{*})$ *denote the firing rate mean and variance at this fixed point*. *Below*, *all derivatives of* *μ* *and* *ν* *with respect to* *a* *and* *b* *are evaluated at* $(a^{*}, b^{*})$.

*The fixed point* $(a^{*}, b^{*})$ *of the averaged system is stable if*

10 $$ \biggl(\frac{\partial\mu}{\partial a}\frac{\partial\nu }{\partial b}-\frac{\partial\mu}{\partial b}\frac{\partial\nu }{\partial a} \biggr) \biggl(\frac{f_{b}''(\mu^{*})}{f_{b}'(\mu^{*})} - \frac {f_{a}''(\mu^{*})}{f_{a}'(\mu^{*})} \biggr) > 0. $$

##### Remark 4

Note that this result is *a*/*b* symmetric: if *a* and *b* are swapped, then the signs of both terms reverse and these sign changes cancel, leaving the stability condition unchanged.

##### Proof

A fixed point $(a^{*}, b^{*})$ of the averaged system is exponentially stable if the Jacobian

$$J = \begin{pmatrix} \frac{\partial F_{a}}{\partial a} & \frac{\partial F_{a}}{\partial b} \\ \frac{\partial F_{b}}{\partial a}& \frac{\partial F_{b}}{\partial b} \end{pmatrix} $$

evaluated at $(a^{*}, b^{*})$ has two negative eigenvalues. By Assumption 2, the Jacobian of the dual control system at $(a^{*}, b^{*})$ has negative trace. A matrix has two negative eigenvalues if it has a negative trace and positive determinant. Therefore, the fixed point $(a^{*}, b^{*})$ of the averaged control system is exponentially stable if

$$\operatorname{det}(J) = \frac{\partial F_{a}}{\partial a}\frac {\partial F_{b}}{\partial b} - \frac{\partial F_{a}}{\partial b}\frac{\partial F_{b}}{\partial a}>0 $$

at $(a^{*}, b^{*})$.

Below, we abbreviate $\mu{(a,b)}$ as *μ* and $\nu{(a,b)}$ as *ν*. In order to write useful expressions for the terms in $\operatorname {det}(J)$, we Taylor-expand $f_{a}(r)$ about *μ* out to second order, writing $f_{a}(r) = f_{a}(\mu) + f'_{a}(\mu)(r - \mu) + \int_{\mu}^{r} (r-s) f_{a}''(s) \,ds$. We similarly expand $f_{b}(r)$ and average these expressions at $(a,b)$ to rewrite the averaged control equations:

$$\begin{aligned} F_{a}(a,b) &= f_{a}(r_{a}) - f_{a}( \mu) - \biggl\langle \int_{\mu}^{r} (r-s) f_{a}''(s) \,ds \biggr\rangle _{(a, b)}, \\ F_{b}(a,b) &= f_{b}(r_{b}) - f_{b}( \mu) - \biggl\langle \int_{\mu}^{r} (r-s) f_{b}''(s) \,ds \biggr\rangle _{(a, b)}. \end{aligned}$$

Differentiating these expressions and evaluating them at $(a^{*}, b^{*})$, we calculate the terms in $\operatorname{det}(J)$:

11 $$ \frac{\partial F_{a}}{\partial b} = - \frac{\partial\mu}{\partial b}f_{a}' \bigl( \mu^{*} \bigr) - \frac{\partial}{\partial b} \biggl\langle \int_{\mu}^{r} (r-s) f_{a}''(s) \,ds \biggr\rangle _{(a, b)}, $$

where all derivatives are evaluated at $(a^{*}, b^{*})$. We have assumed that $f_{a}''(r) \equiv f_{a}''(\mu^{*})$ over the support of $P_{(a^{*}, b^{*})}$, so we can write

$$\begin{aligned} \frac{\partial F_{a}}{\partial b} &= - \frac{\partial\mu}{\partial b}f_{a}' \bigl( \mu^{*} \bigr) - f_{a}'' \bigl(\mu^{*} \bigr) \frac{\partial}{\partial b} \biggl\langle \int_{\mu}^{r} (r-s) \,ds \biggr\rangle _{(a, b)} \\ &= - \frac{\partial\mu}{\partial b}f_{a}' \bigl(\mu^{*} \bigr) - \frac{1}{2}f_{a}'' \bigl(\mu^{*} \bigr) \frac{\partial\nu}{\partial b}. \end{aligned}$$

Likewise for the other three terms $\frac{\partial F_{a}}{\partial a}$, $\frac{\partial F_{b}}{\partial a}$, and $\frac{\partial F_{b}}{\partial b}$. Calculating the determinant of *J* and canceling like terms, we have

12 $$\begin{aligned} \operatorname{det}(J) =& \frac{1}{2}\frac{\partial\mu}{\partial a}f'_{a} \bigl(\mu^{*} \bigr)f_{b}'' \bigl(\mu^{*} \bigr) \frac{\partial\nu}{\partial b} - \frac{1}{2}\frac{\partial\mu }{\partial b}f'_{a} \bigl(\mu^{*} \bigr)f_{b}'' \bigl(\mu^{*} \bigr) \frac{\partial\nu}{\partial a} \\ &{}+ \frac{1}{2}\frac{\partial\mu}{\partial b}f'_{b} \bigl( \mu^{*} \bigr) f_{a}'' \bigl(\mu^{*} \bigr) \frac{\partial\nu}{\partial a} - \frac{1}{2}\frac{\partial\mu}{\partial a}f'_{b} \bigl(\mu^{*} \bigr)f_{a}'' \bigl(\mu^{*} \bigr)\frac{\partial\nu}{\partial b} \end{aligned}$$

13 $$\begin{aligned} =& \frac{1}{2} \biggl(\frac{\partial\mu}{\partial a}\frac{\partial\nu }{\partial b} - \frac{\partial\mu}{\partial b}\frac{\partial\nu }{\partial a} \biggr) \bigl(f'_{a} \bigl(\mu^{*} \bigr)f_{b}'' \bigl(\mu^{*} \bigr) - f'_{b} \bigl(\mu^{*} \bigr) f_{a}'' \bigl(\mu^{*} \bigr) \bigr). \end{aligned}$$

Thus, $(a^{*}, b^{*})$ is exponentially stable if

$$ 0 < \frac{1}{2} \biggl(\frac{\partial\mu}{\partial a}\frac{\partial\nu }{\partial b} - \frac{\partial\mu}{\partial b}\frac{\partial\nu }{\partial a} \biggr) \bigl(f'_{a} \bigl(\mu^{*} \bigr)f_{b}'' \bigl(\mu^{*} \bigr) - f'_{b} \bigl(\mu^{*} \bigr) f_{a}'' \bigl(\mu^{*} \bigr) \bigr) $$

or, equivalently, if

$$ 0 < \biggl(\frac{\partial\mu}{\partial a}\frac{\partial\nu}{\partial b} - \frac{\partial\mu}{\partial b} \frac{\partial\nu}{\partial a} \biggr) \biggl( \frac{f_{b}''(\mu^{*})}{f'_{b}(\mu^{*})} - \frac{f_{a}''(\mu ^{*})}{f'_{a}(\mu^{*})} \biggr). $$

 □

##### Remark 5

In Appendix 2, we drop the assumption that $f_{a}''$ and $f_{b}''$ are constant over the range of *r* and derive a sufficient condition for stability of the form

14 $$ \biggl(\frac{\partial\mu}{\partial a}\frac{\partial\nu }{\partial b}-\frac{\partial\mu}{\partial b}\frac{\partial\nu }{\partial a} \biggr) \biggl(\frac{f_{b}''(\mu^{*})}{f_{b}'(\mu^{*})} - \frac {f_{a}''(\mu^{*})}{f_{a}'(\mu^{*})} \biggr) >\varDelta $$

for a $\varDelta \ge0$ that is close to zero if $f_{a}''$ and $f_{b}''$ do not vary too widely over most of the range of *r*.

##### Remark 6

A similar result to Theorem 2 could be proven for a system with a single slow homeostatic feedback. The limitation of such a system would be in reachability. As the single homeostatic variable *a* changed, the firing rate mean *μ* and variance *ν* could reach only a one-parameter family of values in the $(\mu, \nu)$-space. Thus, most mean/variance pairs would be unreachable. A perturbation to neuronal parameters would displace this one-parameter family in the $(\mu, \nu )$-space, likely making the original mean/variance pair unreachable. Thus, a single homeostatic feedback could only succeed in recovering its original firing rate mean and variance after perturbation in special cases.

By Lemma 1, fixed points of the averaged system that satisfy the criteria for stability under Theorem 2 are stable in the small-*ϵ* limit for the full, un-averaged control system.

## Further Single-Cell Examples

We will focus on examples in which the two homeostatic variables are excitability *x* and synaptic strength *g*, respectively, as discussed before.

The generality of the main results above (which require that $f''_{a}$ and $f''_{b}$ be constant) allows us to investigate a range of different models of firing rate dynamics even if we do not have an explicit expression for the rate distribution *P*. We only need to know dependence of the firing rate mean *μ* and variance *ν* on the control variables to use Theorem 1 to identify control system fixed points and to use Theorem 2 to determine whether those fixed points are stable.

In the more general case addressed in Appendix 2, where the second derivatives are not assumed to be constant, the left side of (10) must be positive *and sufficiently large* to guarantee the existence of a stable fixed point, where the lower bound *Δ* for “sufficiently large” is close to zero if $f''_{a}$ and $f''_{b}$ are nearly constant over most of the distribution $P(r;a,b)$. We will further discuss the simpler case, but all of our analysis can be applied to the more general case by replacing “positive” with “sufficiently large.”

### Returning to Example 1

We can use Theorem 2 to generalize the results for the dually controlled OU process from Sect. 2. We consider the rate model described by the differential equation

15 $$ \tau_{r} \dot{r} = -r +gI{(t)} +{{x}}, $$

where $I{(t)}$ is any second-order stationary process, that is, a process with stationary mean $\phi= \langle I{(t)}\rangle$ and stationary autocovariance function $R(w) = \langle I{(t)} I{(t+w)} \rangle- \phi^{2}$, both independent of *t*. We assume that *r* is ergodic. Let $\mu^{*}$ and $\nu^{*}$ denote the characteristic firing rate mean and variance determined from the parameters of the control system parameters using Theorem 1.

This firing rate process is the output of a stationary linear filter applied to a second-order stationary process, so according to standard results, we have $\mu({{x}},g) = g\phi+{{x}}$ and $\nu({{x}}, g) = \frac {g^{2} C}{2\tau_{r}}$, where $C= \iint_{-\infty}^{t} R(t_{1}-t_{2})e^{-\frac {2t - t_{1} - t_{2}}{\tau_{r}}}\,dt_{1}\,dt_{2}$. Thus, as long as $\nu^{*}>0$, there exists a fixed point of the control system at

$$\bigl({{x}}^{*}, g^{*} \bigr) = \biggl(\mu^{*} - \sqrt{\frac{2\tau_{r}\nu ^{*}}{C}}\phi, \sqrt{ \frac{2\tau_{r}\nu^{*}}{C}} \biggr). $$

At this fixed point, we have $\frac{\partial\mu}{\partial{{x}}} = 1$, $\frac{\partial\mu}{\partial g} = \phi$, $\frac{\partial\nu }{\partial {{x}}} = 0$, and $\frac{\partial\nu}{\partial g} = \frac{g^{*}C}{\tau _{r}}$. This gives us $\frac{\partial\nu}{\partial g}\frac{\partial \mu }{\partial{{x}}}-\frac{\partial\nu}{\partial{{x}}}\frac{\partial \mu }{\partial g}>0$, and the conditions for Theorem 2 are met if $\frac {f_{g }''(\mu^{*})}{f_{g }'(\mu^{*})} -\frac{f_{{{x}} }''(\mu^{*})}{f_{{{x}} }'(\mu^{*})}$ is positive.

**Fig. 3:**
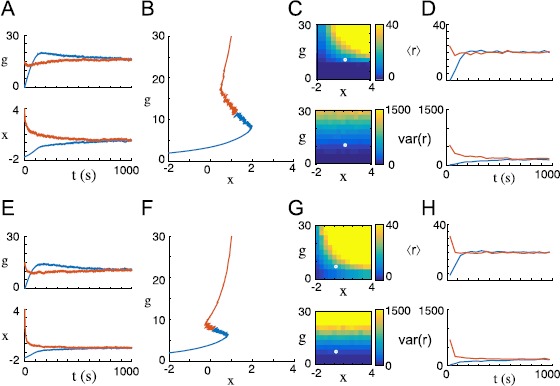
Intrinsic/synaptic dual homeostasis recovers original mean and variance after perturbation in a simulated firing rate model. Firing rate *r* is described by equation (15) with parameter values listed in Appendix 3. Input current is set to $I{(t)} = \phi+ \sigma\xi{(t)}$, where $\xi{(t)}$ is white noise with unit variance. In the top row of figures, $\phi= 0.5$ and $\sigma= 0.25$; in the bottom row of figures, $\phi= 2.5$ and $\sigma= 0.75$. (**A**) *x* and *g* trajectories plotted over time from two different initial conditions (orange and blue) as a fixed point is reached. (**B**) The same trajectories plotted in *x*/*g* phase space. (**C**) Mean firing rate $\langle r \rangle$ (*above*) and firing rate variance $\operatorname{var}(r)$ (*below*) are calculated as a function of homeostatic state $({{x}}, g)$ and represented by color in *x*/*g* parameter space. The fixed point is marked in white. (**D**) Mean firing rate $\langle r \rangle$ (*above*) and firing rate variance $\operatorname{var}(r)$ (*below*) are plotted over time for both initial conditions. (**E**)-(**H**) When the mean and variance of the input current are increased, the system seeks out a new homeostatic fixed point. Note in **G** and **H** that, in spite of the new input statistics, a fixed point is reached with the same firing rate mean and variance

**Fig. 4:**
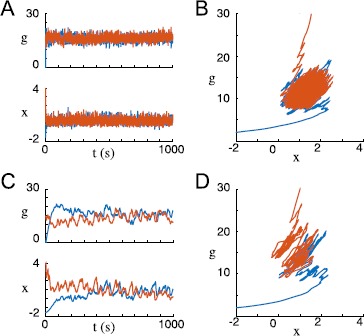
Convergence of intrinsic/synaptic dual homeostasis is compromised by short homeostatic time constants and temporally correlated noise. Firing rate *r* is described by equation (15) with parameter values listed in Appendix 3. (**A**)-(**B**) The system simulated for Fig. 1A-D is modified by reducing homeostatic time constants by a factor of 50: we set $\tau_{{{x}}} = 10~\mbox{s}$ and $\tau_{g} = 1\text{,}000~\mbox{s}$. Trajectories enter and remain within a large neighborhood of the fixed point observed in Fig. 1A, but fluctuate randomly within that neighborhood. By Lemma 1 these trajectories converge in the small-*ϵ* limit, so this neighborhood represents the ball of radius $\alpha(\epsilon)$ that traps all trajectories and shrinks to zero as $\epsilon\rightarrow0$. (**C**)-(**D**) The system described in Fig. 1A is modified by introducing long temporal correlations into the time course of the input current: $I{(t)}$ is an Ornstein-Uhlenbeck process described by the SDE $\tau_{I} \, dI = -I\, dt + d\xi$, where *ξ* is white noise with unit variance and $\tau_{I} = 10~\mbox{s}$. Again, trajectories enter and remain within a large neighborhood of the fixed point observed in Fig. 1A but fluctuate randomly within that neighborhood

Figure 3 shows simulation results for this system under conditions sufficient for stability. Note that if the statistics of the input $I{(t)}$ change, the fixed point changes so that the system maintains its characteristic firing rate mean and variance at equilibrium. In Fig. 4A and Fig. 4B, we alter this system by increasing *ϵ* and by giving $I{(t)}$ correlations on long time scales, respectively. In both these cases, trajectories fluctuate widely about the fixed point of the averaged system but remain within a bounded neighborhood, consistent with the idea of stability in the small-*ϵ* limit.

The slopes $f'_{g }$ and $f'_{{{x}} }$ can be understood as measures of the strength of the homeostatic response to deviations from the target firing rate, and the second derivatives $f''_{g }$ and $f''_{{{x}} }$ can be understood as measures of the asymmetry of this response for upward and downward rate deflections. If *x* and *g* are rescaled to set $f'_{g }\approx f'_{{{x}} }$, then Theorem 2 predicts that dual homeostasis stabilizes a fixed point with a given characteristic mean and variance if $f''_{g }(\mu^{*})>f''_{{{x}} }(\mu^{*})$, thats is, if the (signed) difference between the effects of positive rate deflections and negative rate deflections is greater for the synaptic mechanism than for the intrinsic mechanism.

### Example 2

Intrinsic noise

In Example 1, we assumed that all of the rate fluctuation was due to fluctuating synaptic input. If we introduce an intrinsic source of noise (e.g., channel noise), then the picture becomes slightly more complicated. We set

16 $$ \tau_{r}\dot{r} = -r + g I{(t)} + {{x}}+ \eta\xi{(t)}, $$

where $\xi{(t)}$ is unit-variance white noise independent of $I{(t)}$, and *η* sets the magnitude of the noise. The same calculations as before show that the conditions for stability under Theorem 2 are met at any fixed point for $\frac{f_{g }''(\mu^{*})}{f_{g }'(\mu^{*})} -\frac {f_{{{x}} }''(\mu^{*})}{f_{{{x}} }'(\mu^{*})}>0$. But now the firing rate variance includes the noise variance: $\nu{({{x}}, g)} = \frac{g^{2} C}{2\tau_{r}}+\frac{\eta^{2}}{2\tau_{r}}$. Under Theorem 1, a fixed point only exists if control system parameters are chosen to establish a characteristic variance of $\nu^{*} > \frac{\eta^{2}}{2\tau_{r}}$. This neuron cannot be stabilized with variance less than $\frac{\eta ^{2}}{2\tau _{r}}$ because a variance that low cannot be achieved by the inherently noisy neuron.

**Fig. 5:**
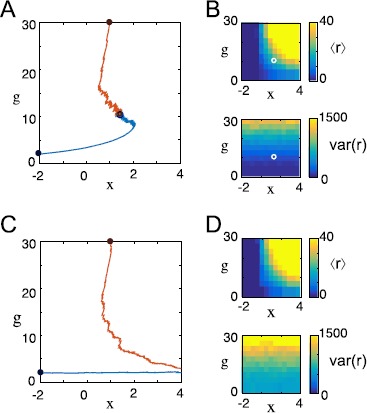
Dual homeostasis tolerates some intrinsic firing rate noise but fails to converge if noise is sufficiently strong. The dynamics of the firing rate *r* are modeled by an intrinsically noisy OU process described by equation (16) with parameter values listed in Appendix 3. (**A**) Intrinsic noise amplitude is set to $\eta= 2$. Dual homeostasis converges on a fixed point near the fixed point of the corresponding system with no intrinsic noise, illustrated in Fig. 1A-D. (**B**) Firing rate mean $\langle r \rangle$ and variance $\operatorname{var}(r)$ are calculated and displayed as functions of *x* and *g* in *x*/*g* parameter space. The fixed point of the system is marked in white. (**C**) Intrinsic noise amplitude is set to $\eta= 10$. Dual homeostasis fails to converge: *x* winds up without bound, and *g* winds down toward zero. (**D**) Note that, due to intrinsic noise, the firing rate variance everywhere in parameter space is larger than the characteristic variance reached at equilibrium in B. Thus, the characteristic variance of this system at equilibrium is unreachable, and dual homeostasis does not converge

In Fig. 5, we show the behavior of this system when $\nu^{*} > \frac{\eta^{2}}{2\tau_{r}}$ (the mean and variance necessary for a fixed point are in the ranges of *μ* and *ν*) and when $\nu^{*} < \frac {\eta ^{2}}{2\tau_{r}}$ (the necessary variance is not in the range of *ν*).

### Example 3

Poisson-spiking neuron with calcium-like firing rate sensor

In some biological neurons, firing rate controls homeostasis via intracellular calcium concentration [4]. Intracellular calcium increases at each spike and decays slowly between spikes, and it activates signaling pathways that cause homeostatic changes. Our dual homeostasis framework is general enough to describe such a system. We let *ρ* represent the concentration of some correlate of firing rate, such as intracellular calcium, and use it in place of firing rate *r*. We model neuronal spiking as a Poisson process with rate $\lambda{(t)} = gI{(t)} + {{x}}$, where $I{(t)}$ is a stationary synaptic input. We let *ρ* increase instantaneously by *δ* at each spike and decay exponentially with time constant $\tau _{d}$ between spikes. We assume that *ρ* is ergodic.

We show in Appendix 4 that, after sufficient time, $y=\rho$ assumes a stationary distribution with mean $\mu{({{x}}, g)} = \delta\tau _{d}(g\phi + {{x}})$ and variance $\nu{({{x}}, g)} = \delta^{2}\tau_{d} (Cg^{2} + \frac{g\phi+{{x}}}{2} )$, where *ϕ* is the stationary mean of $I{(t)}$, and *C* is a positive constant determined by the stationary autocovariance of $I{(t)}$. Thus, we calculate $\frac{\partial\nu }{\partial{{x}}} = \frac{\delta^{2}\tau_{d}}{2}$, $\frac{\partial\mu }{\partial g} = \delta\tau_{d}\phi$, $\frac{\partial\nu}{\partial g} = 2\delta^{2}\tau_{d}Cg^{*} - \frac{\delta^{2}\tau_{d}\phi}{2}$, and $\frac {\partial\mu}{\partial{{x}}} = \delta\tau_{d}$, and we find that $\frac {\partial\nu}{\partial g}\frac{\partial\mu}{\partial{{x}}}-\frac {\partial\nu}{\partial{{x}}}\frac{\partial\mu}{\partial g} = 2\delta ^{3}\tau_{d}^{2}Cg^{*} > 0$. As in Examples 1 and 2, we conclude that the conditions for stability under Theorem 2 are met if $\frac {f_{g }''(\mu ^{*})}{f_{g }'(\mu^{*})} - \frac{f_{{{x}} }''(\mu^{*})}{f_{{{x}} }'(\mu^{*})}>0$.

Note that the conditions for stability in this model are the same as the conditions in the firing rate models. In [8], we show the same result empirically for biophysically detailed model neurons. What all these models have in common is that changes in *g* significantly affect the firing rate variance in the same direction, whereas *x* controls mainly the firing rate mean and has little or no effect on the variance. These results suggest that $\frac{f_{g }''(\mu^{*})}{f_{g }'(\mu^{*})} - \frac{f_{{{x}} }''(\mu^{*})}{f_{{{x}} }'(\mu ^{*})}>0$ is a general, model-independent condition for stability of synaptic/intrinsic dual homeostasis. In Appendix 2, where control function second derivatives are not assumed to be constant, this condition is replaced by the condition of sufficiently large $\frac {f_{g }''(\mu^{*})}{f_{g }'(\mu^{*})} - \frac{f_{{{x}} }''(\mu^{*})}{f_{{{x}} }'(\mu^{*})}$.

As in Example 2, not all mean/variance pairs can be achieved by the control system: no matter how small *g* is, we still have $\nu{({{x}}, g)} \ge\delta^{2}\tau\frac{g\phi+ {{x}}}{2} = \frac{\delta\mu{({{x}}, g)}}{2}$ due to the inherently noisy nature of Poisson spiking, which acts as a restriction on the range of *ν*. We also must have $r>0$, so the range of *μ* is constrained to $\mu{({{x}}, g)}>0$. If $r_{{x}}$ and $r_{g}$ are chosen such that the characteristic firing rate mean $\mu^{*}$ and variance $\nu^{*}$ defined in Theorem 1 obey these inequalities, then there exists a control system state $({{x}}^{*}, g^{*})$ at which Theorem 1 is satisfied and which is therefore a fixed point.

## Recurrent Networks and Integration

A recurrent excitatory network has been shown to operate as an integrator when neuronal excitability and connection strength are appropriately tuned [9, 10]. Such a network can maintain a range of different firing rates indefinitely by providing excitatory feedback that perfectly counteracts the natural decay of the population firing rate. When input causes the network to transition from one level of activity to another, the firing rate of the network represents the cumulative effect of this input over history. Thus, the input is “integrated.”

Below, we show that the parameter values that make such a network an integrator can be stably maintained through dual homeostasis as described before. Importantly, we also show that an integrator network made stable by dual homeostasis is robust to variations in control system parameters and (as in the previous examples) unaffected by changes in input mean and variance. In this section, we build intuition for this phenomenon by investigating a simple example network consisting of one self-excitatory firing rate unit, which may be taken to represent the activity of a homogeneous recurrent network. In Appendix 5, we perform similar analysis for *N* rate-model neurons with heterogeneous parameters. In this case, we do not prove stability, but we do demonstrate that if any neuron’s characteristic variance is sufficiently high, then the network is arbitrarily close to an integrator at any fixed point of the control system.

We consider a single firing rate unit described by the equation

$$ \dot{r} = -r + g \bigl(r + I{(t)} \bigr) +{{x}} + \eta\xi{(t)}, $$

where *η* is the level of intrinsic noise, $I{(t)}$ is a second-order stationary synaptic input with mean *ϕ* and autocovariance $R(w)$, and $\xi{(t)}$ is a white noise process with unit variance. (For simplicity, we have rescaled time to set the time constant $\tau_{r}$ to 1.) Let $m{(t)}$ denote the expected value of *r* at time *t*. Taking the expected values of both sides of the equation, we have

$$ \dot{m} = -m + g (m + \phi) +{{x}}. $$

Let *μ* denote the expected value of *r* once it has reached a stationary distribution. Setting $\dot{m} = 0$, we calculate

17 $$ \mu=\frac{ g \phi+{{x}}}{1-g}. $$

Let $s{(t)}$ denote the deviation of *r* from *m* at time *t*: $s{(t)} := r{(t)} - m{(t)}$. From the equations above we have

18 $$ \dot{s} = -s + g \bigl(s + I{(t)} - \phi \bigr) + \eta\xi {(t)}. $$

If we set $g = 1$, then the *s*-dependence drops out of the right side, and we have

$$ s{(T)} = s{(0)} + \int_{t=0}^{T} \bigl(I{(t)} - \phi+\eta\xi{(t)} \bigr) \,dt. $$

In this extreme case, *s* acts as a perfect integrator of its noise and its input fluctuations, that is, as a noisy integrator. For *g* close to 1, the mean-reversion tendency of *s* is weak, so on short time scales, *s* acts like a noisy integrator.

Next, we write a differential equation for the variance of *r*. From (18) we write

$$ s{(t +dt)} = s{(t)} + dt \bigl[-s{(t)} + g \bigl(s{(t)} + I{(t)} - \phi \bigr) + \eta\xi{(t)} \bigr]. $$

Squaring both sides out to $O(dt)$ and taking the expected value, we have

$$ \bigl\langle s{(t +dt)}^{2} \bigr\rangle = \bigl\langle s{(t)}^{2} \bigr\rangle - 2 \bigl\langle s{(t)}^{2} \bigr\rangle (1-g)\, dt + g^{2} C \,dt + \eta^{2}\,dt, $$

where *C* is a positive constant depending on $\tau_{r}$ and $R(w)$ as in the previous section. Let $\nu:= \lim_{t\rightarrow\infty} \operatorname{var}(r{(t)}) = \lim_{t\rightarrow\infty} \langle s({t})^{2}\rangle$ denote the expected variance of *r* when it has reached a stationary distribution. At this stationary distribution, we have $\langle s{(t +dt)}^{2}\rangle= \langle s{(t)}^{2}\rangle$, so

$$ 0 = - 2(1-g)\nu+ g^{2} C + \eta^{2} $$

or

19 $$ \nu= \frac{1}{2}\frac{ g^{2} C + \eta^{2}}{1-g}. $$

**Fig. 6:**
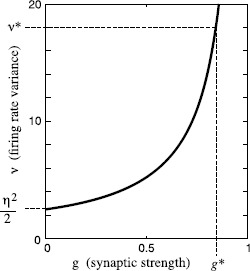
Firing rate variance *ν* vs. synaptic strength *g* in an excitatory recurrent network. Equation (19) is plotted with $\eta^{2} = 5$ and $C = 1$. When synaptic strength is zero, all firing rate variance is due to noise, so $\nu= \frac{\eta^{2}}{2}$. As synaptic strength increases, firing rate variance increases. As synaptic strength approaches unity, recurrent excitation acts to reinforce variations in firing rate, and variance asymptotes to ∞. If target firing rates are set such that the characteristic firing rate variance $\nu^{*}$ is large, then the synaptic strength $g^{*}$ at a control system fixed point must be close to unity, making the network an integrator of its inputs

This relation between *g* and *ν* is plotted in Fig. 6. The right side of this equation is $\frac{\eta^{2}}{2}$ at $g=0$ and increases with *g* until it asymptotes to infinity at $g = 1$. So, given $\nu^{*} > \frac{\eta^{2}}{2}$, there exists exactly one $g^{*}$ at which a firing rate variance of $\nu^{*}$ is achieved. The larger the characteristic variance, the closer $g^{*}$ will be to unity. As discussed before, the firing rate is a good integrator on short time scales if *g* is close to unity. So, given a sufficiently large characteristic variance $\nu^{*}$, the system’s only potentially stable state in the range $0< g^{*}<1$ will allow it to act as a good integrator on short time scales. The larger $\nu^{*}$, the more widely $\nu^{*}$ can be varied while still remaining sufficiently large to make $g^{*}$ close to unity. So, if target firing rates are chosen to make the characteristic variance $\nu^{*}$ sufficiently large, then an integrator achieved in this way is robust to variation in characteristic variance $\nu^{*}$ (and unaffected by variation in $\mu^{*}$).

We can use (17) and (19) to calculate

$$\frac{\partial\nu}{\partial g}\frac{\partial\mu}{\partial {{x}}}-\frac {\partial\nu}{\partial{{x}}}\frac{\partial\mu}{\partial g} = \frac {gC}{1-g} + \frac{\eta^{2}}{2(1-g)^{2}} > 0, $$

so as in the previous examples, the conditions for Theorem 2 are met, and stability of any fixed point is guaranteed if $\frac{f_{g }''(\mu ^{*})}{f_{g }'(\mu^{*})} - \frac{f_{{{x}} }''(\mu^{*})}{f_{{{x}} }'(\mu^{*})}>0$.

In short, if $\frac{f_{g }''(\mu^{*})}{f_{g }'(\mu^{*})} - \frac{f_{{{x}} }''(\mu^{*})}{f_{{{x}} }'(\mu^{*})}>0$ and target rates are chosen to create a sufficiently large characteristic variance $\nu^{*}$, then dual homeostasis of intrinsic excitability and synaptic strength stabilizes a recurrent excitatory network in a state such that the network mean firing rate acts as a near-perfect integrator of inputs shared by the population. The corollary to Theorem 1 tells us that, to first approximation, the characteristic variance is proportionate to the difference between the target rates, and a large characteristic variance is achieved by setting the homeostatic target rates far apart from each other. The integration behavior created in this way is robust to variation in the characteristic mean and variance, and therefore robust to the choice of target firing rates.

This effect can be intuitively understood by noting that as a network gets closer to being a perfect integrator (i.e., as *g* approaches 1), fluctuations in firing rate are reinforced by the resulting fluctuations in excitation. As a result, the tendency to revert to a mean rate grows weaker and the firing rate variance increases toward infinity. (In a perfect integrator, a perturbed firing rate never reverts to a mean, so the variance of the firing rate is effectively infinite.) Thus, the network can attain a large variance by tuning *g* to be sufficiently close to 1. If this large variance is the characteristic variance of the control system, then there is a fixed point of the dual control system at this value of *g*.

In some sense, this behavior is an artifact of the model used—perfect integration is only possible if the feedback perfectly counters the decay of the firing rate over a range of different rates, which is possible in this model because rate increases linearly with feedback and feedback increases linearly with rate. However, such a balance is also achievable with nonlinear rate/feedback relationships if they are locally linear over the relevant range of firing rates. In particular, if the firing rate is a sigmoidal function of input and the eigenvalue of firing rate dynamics near a fixed point is near zero, the upper and lower rate limits act to control runaway firing rates while the system acts as an integrator in the neighborhood of the fixed point. In [8], we show that a recurrent network of biophysically detailed neurons with sigmoidal activation curves can be robustly tuned by dual homeostasis to act as an integrator.

In Appendix 5, we show that integration behavior also occurs at set points in networks of heterogeneous dually homeostatic neurons if one or more of them have a sufficiently large characteristic variance. If only one neuron’s characteristic variance is large, the afferent synapse strength to that neuron grows until that neuron gives itself enough feedback to act as a single-neuron integrator as described before. But if many characteristic variances are large, then all synapse strengths remain biophysically reasonable, and many neurons participate in integration, as might be expected in a true biological integrator network.

**Fig. 7:**
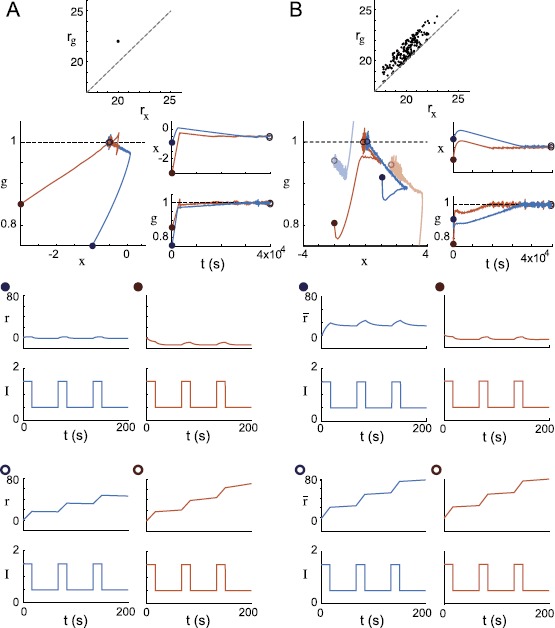
Dual homeostasis creates integrators from a single recurrently excitatory neuron and a heterogeneous excitatory network. (**A**) Dual homeostasis tunes a single neuron with a recurrent excitatory connection to function as an integrator from two different initial conditions (*orange* and *blue*). *First row*: the target rates $r_{{x}}$ and $r_{g}$ are plotted as a point in $(r_{{x}}, r_{g})$ space. *Second row*: the resulting *x* and *g* trajectories are plotted in phase space and over time. *Third row*: before dual homeostasis, the neuron is tested for integrator-like behavior by injecting pulsatile input $I{(t)}$. The firing rate *r* returns to a baseline after each pulse. *Fourth row*: after dual homeostasis, firing rate *r* increases at each pulse and retains its approximate value from one pulse to the next. This neuron is an integrator: its firing rate at any time represents an integral of the pulse history. (**B**) Analogous plots for a heterogeneous recurrently excitatory network of 200 neurons initialized from two initial conditions (*orange* and *blue*). *Top row*: target firing rate pairs of all neurons are plotted in $(r_{{x}}, r_{g})$ space. Note that $r_{g}$ is always chosen to be greater than $r_{{x}}$. *Second row*: average values of *g* and *x* across the network are plotted in phase space and over time. In phase space, a representative trajectory of a single neuron in the network for each of the two simulation runs is also plotted in lighter colors. Note that these trajectories are significantly removed from the average trajectories. *Third row*: both times the network is initialized, the average network rate *r̅* does not act as an integrator for pulsatile input. *Fourth row*: after dual homeostasis, the average network rate acts as an integrator

In Fig. 7, we show simulation results for homogeneous and heterogeneous recurrent networks with target firing rates set to create sufficiently large characteristic firing rate variances. In addition to corroborating our analytical results, these simulations provide empirical evidence that the fixed points of heterogeneous networks are stable under similar conditions to those guaranteeing the stability of the single self-excitatory rate unit discussed before.

## Discussion

This mathematical work is motivated by the observation that the mean firing rates of neurons are restored after chronic changes in input statistics and that this firing rate regulation is mediated by multiple slow biophysical changes [3, 5]. We explore the possibility that these changes represent the action of multiple independent slow negative feedback (“homeostatic”) mechanisms, each with its own constant “target firing rate” at which it reaches equilibrium. Specifically, we focus on a model in which the firing of an unspecified model neuron is regulated by two slow homeostatic feedbacks, which may correspond to afferent synapse strength and intrinsic excitability or any two other neuronal parameters.

In a previous work [8], we showed in numerical simulations that a pair of homeostatic mechanisms regulating a single biophysically detailed neuron can stably maintain a characteristic firing rate mean and variance for that neuron. Here, we have analytically derived mathematical conditions sufficient for any model neuron exhibiting such dual homeostasis to exhibit this behavior. Importantly, the homeostatic system reaches a fixed point when the firing rate mean and variance reach characteristic values determined by homeostasis parameters, so the mean and variance at equilibrium are independent of the details of the neuron model, including stimulus statistics. Thus, this effect can restore a characteristic firing rate mean after major changes in the neuron’s stimulus statistics, as has been observed in vivo, while at the same time restoring a characteristic firing rate variance.

In Theorem 1, we have provided expressions for the characteristic firing rate mean and variance established by a specific set of homeostatic parameters. They show that when the separation between the target rates $r_{a}$ and $r_{b}$ is appropriately small, the relative convexities of the functions $f_{a}$ and $f_{b}$ (by which the firing rate exerts its influence on the homeostatic variables) determine which target rate must be larger for a fixed point to exist. When a fixed point does exist, the characteristic firing rate variance at the fixed point is proportional to the difference between $r_{b}$ and $r_{a}$.

In Theorem 2, we find that any fixed point of our dual homeostatic control system is stable if a specific expression is positive. This expression reflects the mutual influences of firing rate on the homeostatic control system and of control system on the firing rate mean and variance.

Both these theorems are proven under the simplifying assumption that $f''_{a}$ and $f''_{b}$ are constant. However, in Appendices 1 and 2, we drop this assumption and find that qualitatively similar results hold as long as these second derivatives do not vary too widely. In particular, stability is guaranteed if the expression in Theorem 2 exceeds a certain positive bound that is close to zero if $f''_{a}$ and $f''_{b}$ are nearly constant across most of the range of variation of the firing rate.

We have explored the implications of our results for a system with slow homeostatic regulation of intrinsic neuronal “excitability” *x* (an additive horizontal shift in the firing rate curve) and afferent synapse strength *g*. From the corollary to Theorem 1 we find that (to first approximation) stable firing rate regulation requires $r_{g }>r_{{{x}}}$. Using Theorem 2, we show that for rate-based neuron models and Poisson-spiking models, stable firing rate regulation is achieved when the $f_{g }$ is sufficiently concave-up relative to $f_{{{x}}}$.

We predict that these conditions on relative concavity and relative target firing rates should be met by any neuron with independent intrinsic and synaptic mechanisms as its primary sources of homeostatic regulation. Experimental verification of these conditions would suggest that our analysis accurately describes the interaction of a pair of homeostatic mechanisms; experimental contradiction of these conditions would suggest that the control process regulating the neuron could not be accurately described by two independent homeostatic mechanisms providing simple negative feedback to firing rate.

Our results have special implications for neuronal integrators that maintain a steady firing rate through recurrent excitation. We have found that the precise additive and multiplicative tuning necessary to maintain the delicate balance of excitatory feedback can be performed by a dual control system within the framework we study here if the target firing rates are set far enough apart to achieve a large characteristic firing rate variance. The integrator maintained by dual homeostasis is robust to variation of its target firing rates. This occurs because the circuit is best able to achieve a large firing rate variance when it is tuned to eliminate any bias toward a particular firing rate, exactly the condition necessary for integration. This robust integrator tuning scheme should be considered in the ongoing experimental effort to understand processes of integration in the brain.

## Figures and Tables

**Fig. A1:**
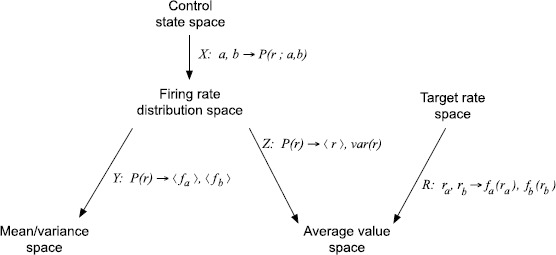
Schematic of mappings defined in Appendix 1

## Appendix 1: Generalization of Theorem 1

Here we present the sense in which the results of Theorem 1 persist for functions $f_{a}$ and $f_{b}$ with nonconstant second derivatives. Mappings defined further are illustrated in Fig. A1.

Let *X* denote the map from a pair $(a,b)$ to the resulting firing rate distribution (with density function $P(\cdot; a,b)$). Let *Y* denote the map from a distribution to its mean and variance $(\mu, \nu)$. Let *Z* denote the map from a distribution to the average values of $f_{a}$ and $f_{b}$ over that distribution. Let *R* denote the map from a pair of firing rate targets $(r_{a},r_{b})$ to $(f_{a}(r_{a}), f_{b}(r_{b}))$, which are the average values of $f_{a}$ and $f_{b}$ necessary for *ȧ* and *ḃ* to average to zero.

For a given set of target rates, the inverse image of $R(r_{a}, r_{b})$ under *Z*, written $Z^{-1}(R(r_{a}, r_{b}))$, is the set of all *r* distributions for which *ȧ* and *ḃ* average to zero. $X^{-1} (Z^{-1}(R(r_{a}, r_{b})) )$ is the set of pairs $(a,b)$ that are fixed points of the averaged control system. $X^{-1} (Y^{-1}(\mu, \nu) )$ is the set of all pairs $(a,b)$ that give rise to distributions with mean *μ* and variance *ν*. Theorem 1, which holds if $f_{a}''$ and $f_{b}''$ are constant, can be written as

$$X^{-1} \bigl(Z^{-1} \bigl(R(r_{a}, r_{b}) \bigr) \bigr) = X^{-1} \bigl(Y^{-1} \bigl( \mu^{*}, \nu^{*} \bigr) \bigr), $$

where $\mu^{*}$ and $\nu^{*}$ are as defined in Theorem 1.

$K_{a}$, $K_{b}$, and the maps *R* and *Z* all change continuously in response to perturbations in $f_{a}$ and $f_{b}$ in the $C^{2}$ space. Therefore, if $f_{a}''$ and $f_{b}''$ are sufficiently close to constants across their domains, the points in $X^{-1} (Z^{-1}(R(r_{a}, r_{b})) )$ are arbitrarily close to points in $X^{-1} (Y^{-1}(\mu^{*}, \nu^{*}) )$ and vice versa. In other words, the fixed points of the control system all give rise to firing rate mean and variance arbitrarily close to $(\mu^{*}, \nu^{*})$, and the control system states that give rise to *r* distributions with firing rate mean and variance of $(\mu^{*}, \nu^{*})$ are arbitrarily close to fixed points.

## Appendix 2: Generalization of Theorem 2

Here we present the sense in which the results of Theorem 2 persist for functions $f_{a}$ and $f_{b}$ with nonconstant second derivatives.

The Jacobian of the averaged control system is continuous with respect to perturbations to $f_{a}$ and $f_{b}$ in $C^{2}$. Therefore, if the determinant of the Jacobian is negative (i.e., a fixed point of the averaged system is stable) for functions $f_{a}$ and $f_{b}$ with constant second derivatives, then when these functions are perturbed in $C^{2}$, the Jacobian is negative at the perturbed fixed point (see the previous appendix).

The persistence of fixed point stability for nonconstant $f_{a}''$ and $f_{b}''$ can be expressed as a generalization of Theorem 2. Rather than requiring an expression to be positive, this generalized theorem requires that the same expression exceed some nonnegative lower bound, which is close to zero if $f_{a}''$ and $f_{b}''$ are sufficiently close to constants over most of the support of the distribution of firing rates *r*.

### Theorem 3

*Let* $(a^{*}, b^{*})$ *denote a fixed point of the averaged control system described before*. *We assume the following*:

1. *The functions* *μ* *and* *ν* *are differentiable at* $(a^{*}, b^{*})$.
2. $\frac{\partial F_{a}}{\partial a}$ *and* $\frac{\partial F_{b}}{\partial b} $ *are negative at* $(a^{*}, b^{*})$, *that is*, *on average*, *a* *and* *b* *provide negative feedback to* *r* *near* $(a^{*}, b^{*})$.

*Let* $\mu^{*} = \mu(a^{*}, b^{*})$ *and* $\nu^{*} = \nu(a^{*}, b^{*})$ *denote the firing rate mean and variance at this fixed point*. *Below*, *all derivatives with respect to* *a* *and* *b* *are evaluated at* $(a^{*}, b^{*})$.

*We define*

$$\begin{aligned} R^{D}_{a/b} =& \sup_{r\in B(\mu^{*}, D)} \bigl\lvert f_{a/b}'' \bigl(\mu^{*} \bigr) - f_{a/b}''(r) \bigr\rvert , \\ \delta^{D}_{a/b} =&1 -\frac{\frac {\partial}{\partial a/b}\nu^{D}}{\frac{\partial}{\partial a/b} \nu}, \\ \nu^{D}{(a,b)} =& \int_{B(\mu^{*}, D)} P(r;a,b) \bigl(r-\mu{(a,b)} \bigr)^{2}\,dr, \\ R_{a/b} =& \liminf_{D\in\mathbb{R}^{+}} \bigl(R^{D}_{a/b} \bigl\lvert 1-\delta^{D}_{a/b} \bigr\rvert + R^{\infty}_{a/b} \bigl\lvert \delta^{D}_{a/b} \bigr\rvert \bigr), \end{aligned}$$

*and*

$$\begin{aligned} \varDelta =& 2 \biggl( \biggl\lvert \frac{\partial\mu}{\partial a}\frac{\partial \nu}{\partial b} \biggr\rvert + \biggl\lvert \frac{\partial\mu}{\partial b}\frac{\partial\nu}{\partial a} \biggr\rvert \biggr) \biggl( \frac {R_{a}}{f'_{a}(\mu^{*})} + \frac{R_{b}}{f'_{b}(\mu^{*})} \biggr) \\ &{}+ \biggl\lvert \frac{\partial\nu}{\partial a} \frac{\partial\nu}{\partial b} \biggr\rvert \frac{\lvert f''_{a}(\mu^{*})\rvert R_{b} + \lvert f''_{b}(\mu ^{*})\rvert R_{a} + R_{a}R_{b}}{f'_{a}(\mu^{*})f'_{b}(\mu^{*})}. \end{aligned}$$

*The fixed point* $(a^{*}, b^{*})$ *of the averaged system is stable if*

20 $$ \biggl(\frac{\partial\mu}{\partial a}\frac{\partial\nu }{\partial b}-\frac{\partial\mu}{\partial b}\frac{\partial\nu }{\partial a} \biggr) \biggl(\frac{f_{b}''(\mu^{*})}{f_{b}'(\mu^{*})} - \frac {f_{a}''(\mu^{*})}{f_{a}'(\mu^{*})} \biggr) > \varDelta . $$

### Remark 7

$R^{D}_{a}$ is the maximal deviation of $f_{a}''(r)$ from $f_{a}''(\mu ^{*})$ on the ball of radius *D*. $\nu^{D}{(a,b)}$ is the amount of variance attributable to probability mass on that ball. Note that $\nu {(a,b)} - \nu^{D}{(a,b)} = \int_{\mathbb{R} \setminus B(\mu^{*}, D)} P(r;a,b)(r-\mu{(a,b)})^{2}\,dr$.

### Proof

We abbreviate $\mu{(a,b)}$ as *μ* and $\nu{(a,b)}$ as *ν*. From (11) we can use the intermediate value theorem to write

$$ \frac{\partial F_{a}}{\partial b} = -\frac{\partial\mu}{\partial b}f_{a}' \bigl( \mu^{*} \bigr) -\frac{\partial}{\partial b} \biggl\langle f_{a}''(c) \int_{\mu}^{r} (r-s) \,ds \biggr\rangle _{(a, b)}, $$

where $c \in[\mu, r]$ depends on *r* and *μ*. Integrating, we have

21 $$ \frac{\partial F_{a}}{\partial b} =- \frac{\partial\mu}{\partial b}f_{a}' \bigl( \mu^{*} \bigr) - \frac{1}{2}\frac{\partial}{\partial b} \bigl\langle f_{a}''(c) (r-\mu)^{2} \bigr\rangle _{(a, b)}. $$

We let

$$J = \begin{pmatrix} \frac{\partial F_{a}}{\partial a} & \frac{\partial F_{a}}{\partial b} \\ \frac{\partial F_{b}}{\partial a}& \frac{\partial F_{b}}{\partial b} \end{pmatrix} . $$

We now define a simpler matrix *J̃* that approximates *J*:

$$\tilde{J} = \begin{pmatrix} X_{aa} & X_{ab} \\ X_{ba} & X_{bb} \end{pmatrix} , $$

where $X_{ab} := -\frac{\partial\mu}{\partial b}f'_{a}(\mu^{*}) - \frac{1}{2}f_{a}''(\mu ^{*})\frac{\partial\nu}{\partial b}$, and likewise for the other three terms in *J̃*. This matrix is identical to the matrix *J* under the assumption of constant $f_{a}''$ and $f_{b}''$. Therefore, from (13) we have

$$\operatorname{det}(\tilde{J}) = \frac{1}{2} \biggl(\frac{\partial\mu }{\partial a} \frac{\partial\nu}{\partial b} - \frac{\partial\mu}{\partial b}\frac{\partial\nu}{\partial a} \biggr) \bigl(f'_{a} \bigl(\mu^{*} \bigr)f_{b}'' \bigl(\mu^{*} \bigr) - f'_{b} \bigl(\mu^{*} \bigr) f_{a}'' \bigl(\mu^{*} \bigr) \bigr). $$

We will estimate how closely the determinant of *J̃* approximates that of *J*. From (21) we write out the expected value as an integral over a probability distribution:

$$ \frac{\partial F_{a}}{\partial b} = -\frac{\partial\mu}{\partial b}f'_{a} \bigl( \mu^{*} \bigr) - \frac{1}{2}\frac{\partial}{\partial b} \int_{\mathbb{R}}P(r;a,b) f_{a}''(c) (r-\mu)^{2} \,dr. $$

Given any radius $D > 0$, we can split the integral into two parts, one on a ball of radius *D* about $\mu^{*}$ and one outside that ball:

$$\begin{aligned} \frac{\partial F_{a}}{\partial b} =& -\frac{\partial\mu}{\partial b}f'_{a} \bigl( \mu^{*} \bigr) - \frac{1}{2} \biggl[\frac{\partial}{\partial b} \int_{B(\mu^{*}, D)}P(r;a,b)f_{a}''(c) (r-\mu)^{2} \,dr \\ &{} + \frac{\partial}{\partial b} \int_{\mathbb{R}\setminus B(\mu^{*}, D)}P(r;a,b)f_{a}''(c) (r-\mu)^{2} \,dr \biggr]. \end{aligned}$$

Applying the intermediate value theorem to both integrals, we have

$$\begin{aligned} \frac{\partial F_{a}}{\partial b} =& -\frac{\partial\mu}{\partial b}f'_{a} \bigl( \mu^{*} \bigr) - \frac{1}{2} \biggl[ \frac{\partial}{\partial b}f_{a}''(c_{1}) \int_{B(\mu^{*}, D)}P(r;a,b) (r-\mu)^{2} \,dr \\ &{}+ \frac{\partial}{\partial b}f_{a}''(c_{2}) \int_{\mathbb{R}\setminus B(\mu^{*}, D)}P(r;a,b) (r-\mu)^{2} \,dr \biggr] \end{aligned}$$

for some $c_{1} \in B(\mu^{*}, D)$ and some $c_{2} \in\mathbb {R}$. By the definition of $\nu^{D}$,

$$ \frac{\partial F_{a}}{\partial b} = -\frac{\partial\mu}{\partial b}f'_{a} \bigl( \mu^{*} \bigr) - \frac{1}{2} \biggl[ f_{a}''(c_{1}) \frac{\partial}{\partial b}\nu^{D} + f_{a}''(c_{2}) \frac{\partial}{\partial b} \bigl(\nu- \nu^{D} \bigr) \biggr]. $$

We compare this quantity to $X_{ab}$:

$$\begin{aligned} \biggl\lvert \frac{\partial F_{a}}{\partial b} - X_{ab} \biggr\rvert =& \frac{1}{2} \biggl\lvert f_{a}''(c_{1}) \frac{\partial\nu^{D}}{\partial b}+ f_{a}''(c_{2}) \frac{\partial}{\partial b} \bigl(\nu- \nu^{D} \bigr) - f_{a}'' \bigl(\mu^{*} \bigr)\frac{\partial\nu}{\partial b} \biggr\rvert \\ =& \frac{1}{2} \biggl\lvert \bigl( f_{a}''(c_{1}) - f_{a}'' \bigl(\mu ^{*} \bigr) \bigr) \frac{\partial\nu^{D}}{\partial b}+ \bigl(f_{a}''(c_{2})- f_{a}'' \bigl(\mu^{*} \bigr) \bigr) \frac{\partial }{\partial b} \bigl(\nu- \nu^{D} \bigr) \biggr\rvert \\ \le& \frac{1}{2} \bigl\lvert f_{a}''(c_{1}) - f_{a}'' \bigl(\mu ^{*} \bigr) \bigr\rvert \biggl\lvert \frac{\partial\nu ^{D}}{\partial b} \biggr\rvert + \frac{1}{2} \bigl\lvert f_{a}''(c_{2})- f_{a}'' \bigl(\mu^{*} \bigr) \bigr\rvert \biggl\lvert \frac{\partial\nu}{\partial b}- \frac{\partial\nu^{D}}{\partial b} \biggr\rvert \\ \le& \frac{1}{2} \biggl\lvert \frac{\partial\nu}{\partial b} \biggr\rvert \biggl(R_{a}^{D} \bigl\lvert 1-\delta_{b}^{D} \bigr\rvert + \frac{1}{2}R_{a}^{\infty}\bigl\lvert \delta_{b}^{D} \bigr\rvert \biggr). \end{aligned}$$

This bound holds for all $D>0$. Taking an infimum over all *D*, we have

22 $$ \biggl\lvert \frac{\partial F_{a}}{\partial b} - X_{ab} \biggr\rvert \le \frac{1}{2} \biggl\lvert \frac{\partial\nu}{\partial b} \biggr\rvert R_{a}. $$

Using this bound (and the equivalent bound for the other three terms of $J - \tilde{J}$), we set bounds on how closely the determinant of *J̃* approximates the determinant of *J*:

$$\begin{aligned} \bigl\lvert \operatorname{det}(J) - \operatorname{det}(\tilde {J}) \bigr\rvert =& \biggl\lvert \biggl(\frac{\partial F_{a}}{\partial a} \frac{\partial F_{b}}{\partial b} - \frac {\partial F_{a}}{\partial b} \frac{\partial F_{b}}{\partial a} \biggr) - (X_{aa}X_{bb} - X_{ab}X_{ba}) \biggr\rvert \\ =& \biggl\lvert \biggl(\frac{\partial F_{a}}{\partial a} \frac {\partial F_{b}}{\partial b} - X_{aa}X_{bb} \biggr) - \biggl(\frac {\partial F_{a}}{\partial b} \frac{\partial F_{b}}{\partial a} - X_{ab}X_{ba} \biggr) \biggr\rvert \\ =& \biggl\lvert \biggl(\frac{\partial F_{a}}{\partial a} - X_{aa} \biggr) \biggl( \frac{\partial F_{b}}{\partial b} - X_{bb} \biggr) \\ &{}+ X_{bb} \biggl( \frac{\partial F_{a}}{\partial a} - X_{aa} \biggr) + X_{aa} \biggl( \frac {\partial F_{b}}{\partial b} - X_{bb} \biggr) \\ &{}- \biggl(\frac{\partial F_{a}}{\partial b} - X_{ab} \biggr) \biggl( \frac{\partial F_{b}}{\partial a} - X_{ba} \biggr) \\ &{}- X_{ba} \biggl( \frac {\partial F_{a}}{\partial b} - X_{ab} \biggr) - X_{ab} \biggl( \frac {\partial F_{b}}{\partial a} - X_{ba} \biggr) \biggr\rvert \\ \le& \biggl\lvert \frac{\partial F_{a}}{\partial a} - X_{aa} \biggr\rvert \biggl\lvert \frac {\partial F_{b}}{\partial b} - X_{bb} \biggr\rvert + \lvert X_{bb} \rvert \biggl\lvert \frac {\partial F_{a}}{\partial a} - X_{aa} \biggr\rvert \\ &{}+ \lvert X_{aa} \rvert \biggl\lvert \frac {\partial F_{b}}{\partial b} - X_{bb} \biggr\rvert + \biggl\lvert \frac{\partial F_{a}}{\partial b} - X_{ab} \biggr\rvert \biggl\lvert \frac {\partial F_{b}}{\partial a} - X_{ba} \biggr\rvert \\ &{} + \lvert X_{ba} \rvert \biggl\lvert \frac {\partial F_{a}}{\partial b} - X_{ab} \biggr\rvert + \lvert X_{ab} \rvert \biggl\lvert \frac {\partial F_{b}}{\partial a} - X_{ba} \biggr\rvert . \end{aligned}$$

From (22) we have

$$\begin{aligned} \bigl\lvert \operatorname{det}(J) - \operatorname{det}(\tilde {J}) \bigr\rvert \le& \frac{1}{2} \biggl\lvert \frac{\partial\nu}{\partial a} \biggr\rvert R_{a}\frac{1}{2} \biggl\lvert \frac{\partial\nu}{\partial b} \biggr\rvert R_{b} + \lvert X_{bb} \rvert\frac {1}{2} \biggl\lvert \frac{\partial\nu}{\partial a} \biggr\rvert R_{a} + \lvert X_{aa} \rvert \frac{1}{2} \biggl\lvert \frac{\partial\nu}{\partial b} \biggr\rvert R_{b} \\ &{}+ \frac{1}{2} \biggl\lvert \frac{\partial\nu}{\partial b} \biggr\rvert R_{a}\frac{1}{2} \biggl\lvert \frac{\partial\nu}{\partial a} \biggr\rvert R_{b} + \lvert X_{ba} \rvert \frac{1}{2} \biggl\lvert \frac{\partial\nu}{\partial b} \biggr\rvert R_{a}+ \lvert X_{ab} \rvert \frac{1}{2} \biggl\lvert \frac{\partial\nu}{\partial a} \biggr\rvert R_{b}. \end{aligned}$$

Substituting the definitions of $X_{aa}$, $X_{ab}$, etc., and simplifying, we get

$$\begin{aligned} \bigl\lvert \operatorname{det}(J) - \operatorname{det}(\tilde {J}) \bigr\rvert \le& f'_{a} \bigl(\mu^{*} \bigr)f'_{b} \bigl(\mu^{*} \bigr) \biggl[ \biggl( \biggl\lvert \frac{\partial\mu }{\partial b} \frac{\partial\nu}{\partial a} \biggr\rvert + \biggl\lvert \frac{\partial\mu}{\partial a} \frac{\partial\nu}{\partial b} \biggr\rvert \biggr) (R_{b} + R_{a} ) \\ &{} + \frac{1}{2} \biggl\lvert \frac{\partial\nu}{\partial a}\frac {\partial\nu}{\partial b} \biggr\rvert \frac{ \lvert f_{a}''(\mu^{*}) \rvert R_{b} + \lvert f_{b}''(\mu ^{*}) \rvert R_{a}+ R_{a}R_{b}}{ f'_{a}(\mu^{*})f'_{b}(\mu^{*})} \biggr] \\ \le& \frac{1}{2}f'_{a} \bigl(\mu^{*} \bigr)f'_{b} \bigl(\mu^{*} \bigr)\varDelta . \end{aligned}$$

Thus, we are guaranteed that $\operatorname{det}(J) > 0$ if $\operatorname{det}(\tilde{J})>\frac {1}{2}f'_{a}(\mu^{*})f'_{b}(\mu^{*})\varDelta $, that is, if

$$\frac{1}{2} \biggl(\frac{\partial\mu}{\partial a}\frac{\partial\nu }{\partial b} - \frac{\partial\mu}{\partial b}\frac{\partial\nu }{\partial a} \biggr) \bigl(f'_{a} \bigl(\mu^{*} \bigr)f_{b}'' \bigl(\mu^{*} \bigr) - f'_{b} \bigl(\mu^{*} \bigr) f_{a}'' \bigl(\mu^{*} \bigr) \bigr)> \frac{1}{2}f'_{a} \bigl(\mu^{*} \bigr)f'_{b} \bigl(\mu^{*} \bigr)\varDelta $$

or, equivalently, if

$$\biggl(\frac{\partial\mu}{\partial a}\frac{\partial\nu}{\partial b}-\frac{\partial\mu}{\partial b} \frac{\partial\nu}{\partial a} \biggr) \biggl(\frac{f_{b}''(\mu ^{*})}{f_{b}'(\mu^{*})} - \frac{f_{a}''(\mu ^{*})}{f_{a}'(\mu^{*})} \biggr) > \varDelta . $$

 □

## Appendix 3: Simulation Parameters

Simulation parameters are given in Tables 1-3.

**Table 1:** **Parameter values in Figs.** **1** **-** **2** **(unless otherwise specified). Parameter values that differed between Figs.** **1** **and** **2** **are separated by a slash.** **Matlab** **function ode45 was used for numerical integration of the averaged control system**

Parameter	Meaning	Value
*ϕ*	Mean input current	1
*σ*	Input current variance	0 / 0.001
$\tau_{{{x}}}$	*x* homeostatic time constant	1
$\tau_{g}$	*g* homeostatic time constant	$\frac{1}{8}$
$r_{{{x}}}$	*x* homeostasis target firing rate	2.5
$r_{g}$	*g* homeostasis target firing rate	3.5
$f_{{{x}}}$	*x* homeostasis control function	$f_{{{x}}}(r) = r$
$f_{g}$	*g* homeostasis control function	$f_{g}(r) = r^{2}$

**Table 2:** **Parameter values in Figs.** **3** **-** **5** **(unless otherwise specified)**

Parameter	Meaning	Value
*dt*	Euler timestep	0.01 s
$\tau_{{{x}}}$	*x* homeostatic time constant	500 s
$\tau_{g}$	*g* homeostatic time constant	50,000 s
$\tau_{r}$	firing rate time constant	0.1 s
$r_{{{x}}}$	*x* homeostasis target firing rate	20
$r_{g}$	*g* homeostasis target firing rate	24
$f_{{{x}}}$	*x* homeostasis control function	$f_{{{x}}}(r) = r$
$f_{g}$	*g* homeostasis control function	$f_{g}(r) = r^{2}$

**Table 3:** **Parameter values in Fig.** **7** **. Parameters that differed between Fig.** **7** **A and Fig.** **7** **B are separated by a slash. In Fig.** **7** **B, for each neuron,** ***ζ*** **is chosen from a normal distribution with mean zero and variance 1, and** ***γ*** **is chosen from a uniform distribution on** $\pmb{[0,2]}$

Parameter	Meaning	Value
*N*	Number of neurons in network	1 / 200
*dt*	Euler timestep	0.01 s / 0.05 s
$\tau_{{{x}}}$	*x* homeostatic time constant	$4\times 10^{4}~\mbox{s}$ / 5,000 s
$\tau_{g}$	*g* homeostatic time constant	$4\times10^{6}~\mbox{s}$ / $1\times10^{6}~\mbox{s}$
$\tau_{r}$	firing rate time constant	1 s
$r_{{{x}}}$	*x* homeostasis target firing rate	20 / 20 + *ζ*
$r_{g}$	*g* homeostasis target firing rate	21 / $r_{{{x}}} + 2\gamma$
$f_{{{x}}}$	*x* homeostasis control function	$f_{{{x}}}(r) = r$
$f_{g}$	*g* homeostasis control function	$f_{g}(r) = r^{2}$

## Appendix 4: Calcium Process Mean and Variance

Here we derive the mean and variance of a process that consists of exponentially decaying impulses arriving as a Poisson process with a rate that varies according to a stationary random input process $I{(t)}$. This process is designed to emulate the dynamics of intracellular calcium, a common sensor of neuronal firing rate, which increases sharply at spike decays slowly between spikes.

Let $I{(t)}$ be a second-order stationary process, that is, a process for which the mean $\langle I{(t)}\rangle$ over instantiations is a constant $\langle I \rangle$ that does not depend on *t*, and the delay-*w* autocovariance $\langle I{(t)}I{(t+w)} \rangle- \langle I \rangle^{2}$ is a function $R(w)$ that does not depend on *t*. Let *ρ* be a positive quantity initialized at zero at $t = 0$ that increases by *δ* at each event of a Poisson process with rate $gI{(t)} + {{x}}$ and decays exponentially with time constant $\tau_{d}$ between events.

We can write

$$\rho{(t)} = \int_{\mathbb{R}^{+}} f(t, s) N(\omega, ds), $$

where $f(t, s) = \mathbf{1}_{s\in[0, t]} \delta e^{-\frac{t-s}{\tau _{d}}}$, and $N(\omega, ds)$ is a Poisson random measure with mean measure $\lambda(ds) = (gI{(s)} +{{x}})\,ds$ [16]. Note that this process is doubly stochastic: $I{(t)}$ is a random process, and $N(\omega, ds)$ is a random measure depending on a specific instantiation of $I{(t)}$. We will write $\langle\cdot\rangle$ to signify the expected value over instantiations of $I{(t)}$, and $\langle\cdot\rangle_{I}$ to signify the expected value given a specific instance of $I{(t)}$. According to [17], the *n*th moment of this process can be written

$$\bigl\langle \bigl(\rho{(t)} \bigr)^{n} \bigr\rangle _{I} = \sum_{(k_{1}, \ldots, k_{n})\in S(n)}K_{n}(k_{1}, \ldots, k_{n})\cdot\prod_{m = 1}^{n} \biggl( \int_{\mathbb{R}} f(t, s)^{m} \lambda(ds) \biggr)^{k_{m}}, $$

where

$$S(n) = \Biggl\{ (k_{1}, \ldots, k_{n})\in\mathbb{N} \times \cdots\times\mathbb{N} \Big| \sum_{i=1}^{n} ik_{i} = n \Biggr\} $$

and

$$K_{n}(k_{1}, \ldots, k_{n}) = \frac{n!}{\prod_{m=1}^{n} m!^{k_{m}} \cdot\prod_{m=1}^{n} k_{m}!}. $$

Thus, for the first moment of $\rho{(t)}$, we have

23 $$\begin{aligned} \bigl\langle \rho{(t)} \bigr\rangle =& \bigl\langle \bigl\langle \rho{(t)} \bigr\rangle _{I} \bigr\rangle \\ =& \biggl\langle \int_{\mathbb{R}} f(t, s) \lambda(ds) \biggr\rangle \\ =& \int_{\mathbb{R}} \mathbf{1}_{s\in[0,t]} \delta e^{-\frac {t-s}{\tau _{d}}} \bigl(g \langle I\rangle+{{x}} \bigr)\,ds \\ =& \delta\tau_{d} \bigl(1 - e^{-\frac{t}{\tau_{d}}} \bigr) \bigl(g\langle I \rangle+{{x}} \bigr)\,ds, \\ \lim_{t\rightarrow\infty} \bigl\langle \rho{(t)} \bigr\rangle =& \delta \tau_{d} \bigl(g\langle I \rangle+{{x}} \bigr)\,ds. \end{aligned}$$

The second moment is given by

$$\begin{aligned} \bigl\langle \bigl(\rho{(t)} \bigr)^{2} \bigr\rangle =& \bigl\langle \bigl\langle \bigl(\rho{(t)} \bigr)^{2} \bigr\rangle _{I} \bigr\rangle \\ =& \biggl\langle \biggl( \int_{\mathbb{R}} f(t, s) \lambda(ds) \biggr)^{2} + \int_{\mathbb{R}} f(t, s)^{2} \lambda(ds) \biggr\rangle \\ =& \biggl\langle \biggl( \int_{\mathbb{R}} \mathbf{1}_{s\in[0,t]} \delta e^{-\frac {t-s}{\tau_{d}}} \bigl(g I{(s)} +{{x}} \bigr) \,ds \biggr)^{2} \biggr\rangle \\ &{}+ \int_{\mathbb{R}} \mathbf{1}_{s\in[0,t]} \delta^{2} e^{-2\frac{t-s}{\tau _{d}}} \bigl\langle \bigl(g I{(s)} +{{x}} \bigr) \bigr\rangle \,ds \\ =& \iint_{s_{1}, s_{2} = 0}^{t} \delta^{2} e^{-\frac {2t-s_{1}-s_{2}}{\tau_{d}}} \bigl\langle \bigl(g I{(s_{1})} +{{x}} \bigr) \bigl(g I{(s_{2})} +{{x}} \bigr) \bigr\rangle \,ds_{1} \,ds_{2} \\ &{}+ \frac{1}{2}\delta^{2}\tau_{d} \bigl(1 - e^{-2\frac{t}{\tau_{d}}} \bigr) \bigl(g\langle I \rangle +{{x}} \bigr). \end{aligned}$$

Setting $w = s_{2} - s_{1}$, we have

$$\begin{aligned} =& \delta^{2} \int_{w=0}^{t} \int_{s_{1} = 0}^{t-w} e^{-\frac {2t-2s_{1}-w}{\tau_{d}}} \bigl\langle \bigl(g I{(s_{1})} +{{x}} \bigr) \bigl(g I{(s_{1}+w)} +{{x}} \bigr) \bigr\rangle \,ds_{1}\,dw \\ &{}+ \frac{1}{2}\delta^{2} \tau_{d} \bigl(1 - e^{-2\frac{t}{\tau_{d}}} \bigr) \bigl(g\langle I \rangle+{{x}} \bigr) \\ =& \delta^{2} \int_{w=0}^{t} \int_{s_{1} = 0}^{t-w} e^{-\frac {2t-2s_{1}-w}{\tau_{d}}} \bigl(g^{2} \bigl\langle I{(s_{1})} I{(s_{1}+w)} \bigr\rangle + 2g \langle I \rangle{{x}} + {{x}}^{2} \bigr) \,ds_{1} \,dw \\ &{}+ \frac{1}{2}\delta^{2}\tau_{d} \bigl(1 - e^{-2\frac{t}{\tau_{d}}} \bigr) \bigl(g\langle I \rangle+{{x}} \bigr) \\ =& \delta^{2} \int_{w=0}^{t} \int_{s_{1} = 0}^{t-w} e^{-\frac {2t-2s_{1}-w}{\tau_{d}}} \bigl(g^{2} \bigl(R(w)+\langle I\rangle^{2} \bigr) + 2g\langle I \rangle{{x}} + {{x}}^{2} \bigr) \,ds_{1} \,dw \\ &{}+ \frac{1}{2} \delta^{2}\tau_{d} \bigl(1 - e^{-2\frac{t}{\tau_{d}}} \bigr) \bigl(g \langle I \rangle+{{x}} \bigr) \\ =& \delta^{2} \int_{w=0}^{t} \frac{\tau_{d}}{2} \bigl(e^{-\frac{w}{\tau_{d}}} - e^{-\frac{2t - w}{\tau_{d}}} \bigr) \bigl(g^{2} R(w) + \bigl(g \langle I\rangle+ {{x}} \bigr)^{2} \bigr) \,dw \\ &{}+ \frac{1}{2}\delta^{2}\tau_{d} \bigl(1 - e^{-2\frac{t}{\tau_{d}}} \bigr) \bigl(g\langle I \rangle+{{x}} \bigr) \\ =& \frac{1}{2}\delta^{2} \tau_{d} g^{2} X_{t} + \frac{1}{2}\delta^{2} \tau_{d}^{2} \bigl(g \langle I\rangle+ {{x}} \bigr)^{2} \bigl(1 - e^{-\frac {t}{\tau_{d}}} \bigr)^{2} \\ &{}+ \frac {1}{2}\delta^{2}\tau_{d} \bigl(1 - e^{-2\frac{t}{\tau_{d}}} \bigr) \bigl(g\langle I \rangle +{{x}} \bigr), \end{aligned}$$

where $X{(t)} = \int_{w=0^{+}}^{t} (e^{-\frac{w}{\tau_{d}}} - e^{-\frac{2t - w}{\tau_{d}}})R(w)\,dw$. Substituting from (23), we have

$$\begin{aligned} \operatorname{var} \bigl(\rho{(t)} \bigr) =& \bigl\langle \bigl(\rho{(t)} \bigr)^{2} \bigr\rangle - \bigl\langle \rho{(t)} \bigr\rangle ^{2} \\ =& \frac{1}{2}\delta^{2} \tau_{d} g^{2} X{(t)} + \frac{1}{2}\delta^{2} \tau_{d} \bigl(1 - e^{-2\frac{t}{\tau_{d}}} \bigr) \bigl(g\langle I \rangle+{{x}} \bigr), \\ \lim_{t\rightarrow\infty}\operatorname{var} \bigl(\rho{(t)} \bigr) =& \frac{1}{2}\delta^{2} \tau_{d} \bigl[ g^{2} X{(\infty)} + \bigl(g\langle I \rangle+{{x}} \bigr) \bigr]. \end{aligned}$$

## Appendix 5: Recurrent Networks with Heterogeneity

Here we show that some of our results on homogeneous recurrent excitatory networks generalize to heterogeneous networks in which each neuron has its own dual control system, target rates, and level of intrinsic noise. In particular, we show that if the neurons in such a network have target firing rates that set at least one of the characteristic variances sufficiently high, then when the combined control system reaches a fixed point, the network behaves like an integrator on short time scales. This condition is robust to variation in individual neuronal parameters, including target firing rates. Since the combined control systems of a heterogeneous network with *N* neurons is 2*N*-dimensional rather than two-dimensional, we do not attempt to prove the stability of fixed points here. However, we have verified the stability of the behavior described below in simulations (data not shown, code available online).

We consider a collection of *N* neurons with firing rates $r_{[1]}, \ldots, r_{[N]}$ connected all-to-all by excitatory synapses. Each neuron *n* has its own homeostatic variables ${{x}}_{[n]}$ and $g_{[n]}$, its own extrinsic second-order stationary synaptic input $I{(t)}_{[n]}$ with mean $\phi_{[n]}$ and autocovariance $R_{[n]}(w)$, its own intrinsic noise $\eta_{[n]}\xi_{[n]}{(t)}$ (where $\xi _{[n]}{(t)}$ is a white noise process with unit variance), a second-order stationary synaptic input $I_{\mathrm{all}}{(t)}$ with mean $\phi _{\mathrm{all}}$ and autocovariance $R_{\mathrm{all}}(w)$ shared by all neurons, and excitatory synaptic input from all other neurons, which together determine the firing rate as in the rate models above. Each neuron *n* has its own target firing rates $r_{{{x}} [n]}$ and $r_{g [n]}$. Each neuron *n* delivers an additional excitatory synaptic input $\frac {1}{N}r_{[n]}$ to each other neuron.

We write the rate model for neuron *n* in the form

$$\begin{aligned} r_{[n]}{(t+dt)} =& r_{[n]}{(t)} + dt \biggl[-r_{[n]}{(t)} + g_{[n]}I_{[n]}{(t)} + g_{[n]}I_{\mathrm{all}}{(t)} \\ &{}+ g_{[n]}\sum_{m}\frac {1}{N}r_{[m]}{(t)} + {{x}}_{[n]} + \eta_{[n]}\xi_{[n]}{(t)} \biggr]. \end{aligned}$$

Here we have rescaled time in order to ignore the time constant $\tau _{r}$. We can take expected values of both sides to describe the expected firing rate at time *t*, $\mu_{[n]}{(t)} := \langle r_{[n]}{(t)}\rangle$:

24 $$\begin{aligned} \begin{aligned}[b] \mu_{[n]}{(t+dt)} ={}& \mu_{[n]}{(t)} + dt \biggl[-\mu _{[n]}{(t)} + g_{[n]}\phi_{[n]} + g_{[n]} \phi_{\mathrm{all}} \\ &{}+ g_{[n]}\sum_{m} \frac{1}{N}\mu_{[m]}{(t)} + {{x}}_{[n]} \biggr]. \end{aligned} \end{aligned}$$

Let $s_{[n]}{(t)}$ denote the deviation of rate *n* from its expected value: $s_{[n]}{(t)} := r_{[n]}{(t)} - \mu_{[n]}{(t)}$. Taking the difference of the last two equations, we have

25 $$\begin{aligned} s_{[n]}{(t+dt)} =& s_{[n]}{(t)} + dt \biggl[-s_{[n]}{(t)} + g_{[n]} \bigl(I_{[n]}{(t)}-\phi_{[n]} \bigr) + g_{[n]} \bigl(I_{\mathrm{all}}{(t)}-\phi_{\mathrm{all}} \bigr) \\ &{} + \frac{1}{N}g_{[n]}\sum_{m}s_{[m]}{(t)} + \eta_{[n]}\xi_{[n]}{(t)} \biggr]. \end{aligned}$$

Let $\mathbf{r}{(t)} := \frac{1}{N}\sum_{m}r_{[m]}{(t)}$ denote the average network firing rate at time *t*. The expected value of $\mathbf {r}{(t)}$ is $\frac{1}{N}\sum_{m}\mu_{[m]}{(t)}$. Let $\mathbf {s}{(t)}:=\mathbf{r}{(t)} - \frac{1}{N}\sum_{m}\mu_{[m]}{(t)} = \frac {1}{N}\sum_{m}s_{[m]}{(t)}$ denote the deviation of $\mathbf{r}{(t)}$ from its expected value. Taking the average over *n* in (25), we have

26 $$\begin{aligned} \mathbf{s} {(t+dt)} =& \mathbf{s} {(t)} + dt \biggl[- \biggl(1 - \frac {1}{N}\sum_{n} g_{[n]} \biggr) \mathbf{s} {(t)}+ \frac{1}{N}\sum_{n} g_{[n]} \bigl(I_{[n]}{(t)}-\phi_{[n]} \bigr) \\ &{} + \frac{1}{N}\sum_{n} g_{[n]} \bigl(I_{\mathrm{all}}{(t)}-\phi_{\mathrm{all}} \bigr) + \frac {1}{N}\sum _{n}\eta_{[n]}\xi_{[n]}{(t)} \biggr]. \end{aligned}$$

Let $\overline{g} := \frac{1}{N}\sum_{n} g_{[n]}$. For $\overline{g} <1$, $\mathbf{s}{(t)}$ is a mean-zero OU process. If we set $\overline{g} = 1$, then the term $- (1 - \frac{1}{N}\sum_{n} g_{[n]} )\mathbf {s}{(t)}$ drops out, and we can solve for $\mathbf{s}{(t)}$:

27 $$\begin{aligned} \mathbf{s} {(T)} =& \mathbf{s} {(0)} + \int_{t=0}^{T} \,dt \biggl[\frac{1}{N}\sum _{n} g_{[n]} \bigl(I_{[n]}{(t)}- \phi_{[n]} \bigr) \\ &{}+ \frac{1}{N}\sum_{n} g_{[n]} \bigl(I_{\mathrm{all}}{(t)} -\phi_{\mathrm{all}} \bigr)+ \frac{1}{N}\sum_{n}\eta_{[n]}\xi _{[n]}{(t)} \biggr]. \end{aligned}$$

In this case, the mean network firing rate acts as a perfect integrator of all of the network’s input fluctuations and noise. The closer *g̅* is to 1, the more $\mathbf{s}{(t)}$ acts like an integrator on short time scales.

For $\overline{g}<1$, the vector of rates $r_{[n]}{(t)}$ is a linearly filtered second-order stationary random process and therefore approaches a stationary mean and variance. Using (25), we write a recursive equation for the covariance of $s_{[n]}{(t)}$ and $s_{[m]}{(t)}$ out to $O(dt)$:

$$\begin{aligned} &\bigl\langle s_{[n]}{(t+dt)}s_{[m]}{(t+dt)} \bigr\rangle \\ &\quad = \bigl\langle s_{[n]}{(t)}s_{[m]}{(t)} \bigr\rangle - 2 \bigl\langle s_{[n]}{(t)}s_{[m]}{(t)} \bigr\rangle \,dt \\ &\qquad {} + \frac{1}{N} \biggl[g_{[m]}\sum _{j} \bigl\langle s_{[n]}{(t)}s_{[j]}{(t)} \bigr\rangle + g_{[n]}\sum_{j} \bigl\langle s_{[m]}{(t)}s_{[j]}{(t)} \bigr\rangle \biggr]\,dt \\ &\qquad {}+ g_{[n]}g_{[m]}C_{\mathrm{all}} \,dt + \delta_{n=m} \bigl[g_{[n]}^{2}C_{[n]} + \eta _{[n]}^{2} \bigr]\,dt, \end{aligned}$$

where $C_{\mathrm{all}}$ and $C_{[n]}$ are constants depending on the input autocovariance functions, and $\delta_{n=j}$ is 1 if $n=j$ and zero if $n\neq j$. Once all rates have reached a stationary joint distribution, we should have $\langle s_{[n]}{(t+dt)}s_{[m]}{(t+dt)} \rangle = \langle s_{[n]}{(t)}s_{[m]}{(t)} \rangle$, so from the last equation we have

$$\begin{aligned} 0 =& - 2 \bigl\langle s_{[n]}{(t)}s_{[m]}{(t)} \bigr\rangle + \frac{1}{N} \biggl[g_{[m]}\sum_{j} \bigl\langle s_{[n]}{(t)}s_{[j]}{(t)} \bigr\rangle + g_{[n]}\sum_{j} \bigl\langle s_{[m]}{(t)}s_{[j]}{(t)} \bigr\rangle \biggr] \\ &{} + g_{[n]}g_{[m]}C_{\mathrm{all}} + \delta_{n=m} \bigl[g_{[n]}^{2}C_{[n]} + \eta_{[n]}^{2} \bigr]. \end{aligned}$$

Thus, the stationary covariances $\langle s_{[n]}{(t)}s_{[m]}{(t)} \rangle$ are related by the equation

28 $$\begin{aligned} \bigl\langle s_{[n]}{(t)}s_{[m]}{(t)} \bigr\rangle =& \frac{1}{2N} \biggl[g_{[m]}\sum_{j} \bigl\langle s_{[n]}{(t)}s_{[j]}{(t)} \bigr\rangle + g_{[n]}\sum_{j} \bigl\langle s_{[m]}{(t)}s_{[j]}{(t)} \bigr\rangle \biggr] \\ &{} + \frac{1}{2}g_{[n]}g_{[m]}C_{\mathrm{all}} + \frac{1}{2}\delta_{n=m} \bigl[g_{[n]}^{2}C_{[n]} + \eta_{[n]}^{2} \bigr]. \end{aligned}$$

Summing this expression over *n* and *m*, we have

29 $$\begin{aligned} \sum_{n,m} \bigl\langle s_{[n]}{(t)}s_{[m]}{(t)} \bigr\rangle =& \overline{g} \sum_{n,m} \bigl\langle s_{[n]}{(t)}s_{[m]}{(t)} \bigr\rangle + \frac{1}{2} \biggl(\sum_{n}g_{[n]} \biggr)^{2}C_{\mathrm{all}} \\ &{}+ \frac {1}{2}\sum _{n} g_{[n]}^{2}C_{[n]} + \frac{1}{2}\sum_{n} \eta_{[n]}^{2}. \end{aligned}$$

We set $\varOmega:= \frac{1}{N^{2}} \sum_{n,m} \langle s_{[n]}{(t)}s_{[m]}{(t)} \rangle$. Solving for *Ω*, we have

30 $$ \varOmega= \frac{1}{2}\frac{\overline{g}^{2}C_{\mathrm{all}} + \frac {1}{N^{2}}\sum_{m} g_{[m]}^{2}C_{[m]} + \frac{1}{N^{2}}\sum_{m} \eta _{[m]}^{2}}{1-\overline{g}}. $$

We sum over *m* in (28) and write the result in terms of *Ω*:

31 $$\begin{aligned} \sum_{m} \bigl\langle s_{[n]}{(t)}s_{[m]}{(t)} \bigr\rangle =& \frac{1}{2}\overline{g}\sum_{j} \bigl\langle s_{[n]}{(t)}s_{[j]}{(t)} \bigr\rangle + \frac{N}{2}g_{[n]}\varOmega \\ &{}+ \frac {1}{2}g_{[n]} \overline{g}C_{\mathrm{all}} + \frac{1}{2}g_{[n]}^{2}C_{[n]} + \frac {1}{2}\eta_{[n]}^{2}. \end{aligned}$$

We set $\varSigma_{[n]} := \frac{1}{N}\sum_{m} \langle s_{[n]}{(t)}s_{[m]}{(t)} \rangle$ and solve for $\varSigma_{[n]}$:

32 $$ \varSigma_{[n]} = \frac{g_{[n]} \varOmega+ \frac {1}{N}g_{[n]}\overline{g}C_{\mathrm{all}} + \frac {1}{N}g_{[n]}^{2}C_{[n]} + \frac {1}{N}\eta_{[n]}^{2}}{2-\overline{g}}. $$

Finally, from (28) we have

33 $$ \bigl\langle \bigl( s_{[n]}{(t)} \bigr)^{2} \bigr\rangle = F_{[n]}(\mathbf{g}) = g_{[n]}\varSigma_{[n]} + \frac {1}{2}g_{[n]}^{2}C_{\mathrm{all}} + \frac{1}{2}g_{[n]}^{2}C_{[n]} + \frac{1}{2}\eta_{[n]}^{2}, $$

where **g** is the vector of all $g_{[m]}$.

Suppose that target firing rates are set such that each neuron *n* has characteristic variance $\nu_{[n]}^{*}$. Then at any fixed point $(\mathbf {{{x}}}, \mathbf{g})$ of the combined control system, we have $\nu _{[n]}^{*} = F_{[n]}(\mathbf{g})$ for all *n*. It is easy to check that $F_{[n]}(\mathbf{g})$ is $\frac{1}{2}\eta_{[n]}^{2}$ at $g_{[n]}=0$, that it increases with any with respect to any $g_{[m]}$ on the domain $\{ \sum_{m} g_{[m]} < N\} \cap\{g_{[n]}>0\}$, and that it asymptotes to infinity as $\overline{g} = \frac{1}{N}\sum_{m} g_{[m]}$ approaches 1. Thus, for sufficiently large $\nu_{[n]}^{*}$, the constraint $\nu_{[n]}^{*} = F_{[n]}(\mathbf{g})$ can be solved on the domain, and any solution must be close to the hypersurface $\overline{g} = 1$. As discussed before, the system acts as an integrator on short time scales when *g̅* is close to 1; thus, if any one or more of the characteristic variances $\nu_{[n]}^{*}$ are sufficiently large, then at a fixed point of the combined control system, the mean network firing rate acts as an integrator on short time scales.

We can intuitively see that if only one neuron, neuron *n*, has a high characteristic variance, then it acts like the single-neuron integrator as $g_{[n]}$ approaches *N*, and neuron *n* dominates the global feedback signal. This scenario is not particularly biophysically interesting. However, if many neurons have high characteristic variance, then no individual neuron will dominate the feedback signal. In the special case that all neuron parameters are identical, a symmetry argument shows that $g_{[n]}$ will be close to 1 for all *n*—no single synapse is strong enough to make a single neuron an integrator, but the fluctuations in network firing rate cause correlated fluctuations in the individual firing rates, which collectively reinforce the network rate fluctuations, causing the network as a whole act as an integrator.

By the corollary of Theorem 1, the characteristic variance of a neuron is proportionate to the difference between target firing rates as long as this difference remains small on the scale of the convexity of $f_{a}$ and $f_{b}$; so if $f_{a}''$ and $f_{b}''$ are small, then a large characteristic variance can be achieved by setting target firing rates sufficiently far apart, and perturbations to this difference cause proportionate perturbations in characteristic variance. If the characteristic variances $\nu_{[n]}^{*}$ are sufficiently large to make the network an integrator, then they will remain sufficiently large even in the face of such perturbations. We conclude that a network integrator stabilized by dual homeostasis is robust to perturbations in the target firing rates $r_{{x}}$ and $r_{g}$.
